# Severe mental illness and the risk of breast cancer: A two-sample, two-step multivariable Mendelian randomization study

**DOI:** 10.1371/journal.pone.0291006

**Published:** 2023-09-01

**Authors:** Yongjia Cui, Wenping Lu, Tianrui Shao, Zhili Zhuo, Ya’nan Wang, Weixuan Zhang

**Affiliations:** 1 Oncology Department, China Academy of Chinese Medical Sciences Guang’anmen Hospital, Beijing, China; 2 First Teaching Hospital of Tianjin University of Traditional Chinese Medicine, National Clinical Research Center for Chinese Medicine Acupuncture and Moxibustion, Tianjin, China; Iranian Institute for Health Sciences Research, ISLAMIC REPUBLIC OF IRAN

## Abstract

**Background:**

Based on epidemiological reports, severe mental illness (SMI) and breast cancer (BC) risk are linked positively. However, it is susceptible to clinical confounding factors, such as smoking, alcohol consumption, etc. Here, we performed a two-sample, two-step multivariable Mendelian randomization (MR) research to explore how the SMI etiologically influences BC risk and to quantify mediating effects of known modifiable risk factors.

**Methods:**

Data concerning the single nucleotide polymorphism (SNP)-associated with schizophrenia, bipolar disorder (BD), major depressive disorder (MDD), and BC were obtained from two large consortia: the Breast Cancer Association Consortium (BCAC) and the Psychiatric Genomics Consortium (PGC). Then, the correlations of the previous SMI with the BC prevalence and the potential impact of mediators were explored through the two-sample and two-step MR analyses.

**Results:**

In two-sample MR, schizophrenia increased BC incidence (odds ratio (OR) 1.06, 95% confidence interval (CI) 1.02–1.10, P = 0.001). In subgroup analysis, schizophrenia increased ER+ BC (OR 1.06, 95% CI 1.03–1.10, P = 0.0009) and ER-BC (OR 1.06, 95% CI 1.01–1.11, P = 0.0123) incidences. Neither MDD nor BD elevated the BC risk. In two-step MR, smoking explained 11.29% of the schizophrenia-all BC risk association.

**Conclusions:**

Our study indicates that schizophrenia increases susceptibility to breast cancer, with smoking playing a certain mediating role. Therefore, BC screening and smoking should be incorporated into the health management of individuals with schizophrenia.

## Introduction

The number of individuals afflicted by severe mental illness (SMI) is increasing annually [1]. Those with SMI, including schizophrenia, bipolar disorder (BD), and major depressive disorder (MDD), experience a life expectancy reduced by 10–20 years compared to the general population [2]. The occurrence of cancers, including breast cancer (BC), exacerbates this shortened lifespan. Research shows that after adjusting for age, income, race, ethnicity, geographic location, and marital status, individuals with SMI face a twofold increased risk in all-cause mortality compared to those without mental disorders [3]. Newly diagnosed female BC cases are more significant than the corresponding cases of lung carcinomas, depicting the leading position of BC in worldwide carcinoma incidence [4]. Early diagnosis of BC can improve the prognosis [5]. However, BC patients with SMI are more susceptible to being diagnosed with advanced BC and invasive tumor characteristics due to a lack of regular BC screenings [6]. This highlights the importance of focusing on BC health management among individuals with severe mental disorders.

Emotional abnormalities and psychological stress will affect the occurrence and metastasis of BC through various mechanisms [7, 8]. In addition, evidence reveals that patients with SMI have fewer BC screenings [9] and less adherence to anticancer therapy [10], making BC treatment challenging in patients with SMI. Therefore, clarifying the linkage of SMI to the incidence of BC seems imperative, which can heighten awareness among patients and healthcare professionals, leading to early detection and treatment, thus enhancing BC survival rates. Clinical studies depict an elevated BC risk among the SMI population. However, they will be affected by clinical confounding factors, such as smoking [11], obesity [12], long-term use of anti-schizophrenic drugs [13], diabetes [14], physical activity [15], and alcohol consumption [16].

Mendelian randomization (MR) has been widely utilized in causality inference [17, 18]. A two-sample MR is one of the essential strategies in which the single nucleotide polymorphism (SNP)-exposure and outcomes are acquirable based on two genome-wide association studies (GWAS) without overlapping samples, increasing the applicability of MR. Utilizing the summary data from two GWAS studies, evaluating the causal influence of exposure on the outcomes is possible. This is a huge advantage, allowing the performance of causal inferences between two traits even if their measurements are not from an identical sample set. It will enable us to take full advantage of statistics from various existing GWAS studies. The two-step MR, based on a two-sample MR strategy, investigates the possible roles of mediators between the exposure and the outcomes.

In this study, we investigated the causal relationship between SMI and the incidence of BC using two-sample Mendelian randomization (MR), including Schizophrenia, major depressive disorder (MDD), and bipolar disorder (BD). Additionally, we employed a two-step MR approach to explore the mediating effects of smoking initiation, alcohol consumption, physical activity, type 2 diabetes, prolactin levels, and the body mass index (BMI) in the causal link between SMI and BC. The inverse-variance weighted (IVW) method was utilized as the primary algorithm to estimate the magnitude of causal effects. Understanding the association between SMI and BC occurrence could aid in enhancing BC screening and prevention among SMI patients, contributing to more effective health management strategies for individuals with SMI.

## Materials and methods

### Study design

This work is an Mendelian randomization (MR) research, with a two-sample MR and a two-step MR, whose implementation relied on three hypotheses: (1) There exist robust correlations of instrumental variables (IVs) with the exposure (association); (2) IVs are irrespective of the confounders (independence); (3) the effect of IVs on outcomes is achievable solely through exposure (exclusion restriction criteria).

All the data comes from online databases. This study involves a reanalysis of publicly available GWAS data. All included studies have received approval from their respective academic ethics review committees, and written informed consent has been obtained from each participant. Therefore, no additional ethical approval is required.

### Genetic instrumental variables for exposures

Summary data regarding the associations of schizophrenia [19] BD [20] and MDD [21], with single nucleotide polymorphisms (SNPs) were gathered from the Psychiatric Genomics Consortium (PGC) (https://www.med.unc.edu/pgc/), which is one of the largest, most innovative, and most productive consortia within the psychiatric domain. The PGC analyzes the genetic characteristics of schizophrenia, BD, MDD, and other mental diseases. A GWAS research [19] is the source of summary data from schizophrenia-associated SNPs, which comprises up to 36989 cases and 113075 controls. A large GWAS meta-analysis [20] provided summary data for BD-related SNPs, comprising 20,352 cases and 31,358 controls. Meanwhile, the most significant GWAS meta-analysis [21] depicted MDD-related SNPs, including the three most extensive depression-related studies [22–24] involving 246,363 cases and 561,190 controls (after elimination of overlapping samples), amounting to 807,553 subjects.

We selected SNPs with p < 5e-08 and removed linkage disequilibrium (LD) (r^2^ < 0.001, distance threshold = 10000 kb) to guarantee a strong association of SNPs with exposures. In addition, the F-statistics exceed 10 for the selected SNPs, indicating that the instrumental variables are associated powerfully with the exposure [25]. The computational formula for F-statistic herein is Beta^2^/SE^2^, where Beta refers to the genetic correlation with exposure and SE stands for standard deviation. We selected 147 SNPs as instrumental variables (S1 Table in S1 Data).

### Genetic instrumental variables for potential mediators

The mediators-associated SNPs came from the GWAS summary data(https://gwas.mrcieu.ac.uk/), including smoking initiation, alcohol consumption, physical activity, type 2 diabetes, prolactin levels, and the body mass index (BMI) (S2 Table in S1 Data). The smoking initiation related-SNPs were obtained from the GSCAN, a worldwide consortium of genetic correlation meta-analyses, including up to 1.2 million individuals. The Within Family Consortium was the source of alcohol consumption-related-SNPs, consisting of researchers intending to comprehend the effects of the human genome on a broad spectrum of socioeconomic and medical traits based on the relevant individual datasets. The UK Biobank was the source of physical activity related-SNPs. The largest GWAS of physical activity was performed with three self-report-based measures and two wrist-worn accelerometry data-based measures. The type 2 diabetes and BMI-related-SNPs were obtained from the MRC Integrative Epidemiology Unit. A genome-wide meta-analysis was the source of prolactin levels related to SNPs, involving 21,758 participants.

### Genetic instrumental variables for BC

The source of pooled data on BC-associated SNPs with 122,977 cases (69,501 ER+ cases plus 21,468 ER cases) and 105,974 controls was the Breast Cancer Association Consortium (BCAC) [26]. We performed subgroup analyses based on the status of estrogen expression.

### MR analyses

A two-sample MR analysis exploring the exposures’ impact on BC employs three analytical approaches: the IVW (inverse-variance weighted; the foremost approach), the MR-Egger, and the weighted median methods. During the MR analysis, the IVW achieves meta-aggregation of the multiple loci’s effects (beta0). The application principle of IVW is that the SNPs are entirely irrespective of each other and are valid IVs [27]. MR-Egger regression is a method for achieving pleiotropy identification and adjustment in the MR analysis, relaxing the requirement that there is no pleiotropy between genetic variants within IVW methods. In the case of MR-Egger regression, its intercept estimates become the mean of pleiotropic effect estimates within each genetic variant [28]. With the weighted median approach, the medians are derived through weight-dependent sorting of all the independent values within the SNP effect. The weighted median can become a robust estimate if the MR core hypotheses are satisfied by a minimum of 50% genetic variation [29].

In the first step of the two-step MR for the effect mediators, the causal effect of exposure to the mediator variable (beta1) was calculated using the two-sample MR method. The second step involves estimating the mediator’s causal influence on the outcomes (beta2) by the multivariate MR method. Beta0, beta1, and beta2 were all significant, indicating a causal relationship between exposure and outcome, and mediator variables could partly mediate this relationship. In general, we can take beta1*beta2 as the mediating effect from exposure to the outcome and beta1*beta2/beta0 as the proportion of the mediating effect within the causal relationship [30]. Beta0 was not significant, but both beta1 and beta2 were significant, suggesting that this mediating variable entirely mediated the association from exposure to outcome. Beta0 is significant, but at least one of beta1 and beta2 is not significant, indicating that this mediating variable has no effect on the causal association from exposure to the outcome.

### Sensitivity analysis

The causal effect estimates were biased due to the possible heterogeneity among IVs. Our study used the Cochran Q statistic of the MR-Egger and the IVW method to perform a heterogeneity test for detecting heterogeneity. P > 0.05 indicated no heterogeneity in IV. Therefore, we applied a fixed-effects model in the MR analysis; otherwise, a random-effects model was utilized.

Confounders are probable mediators of the exposure-outcome causality. We tested the horizontal pleiotropy of IVs by utilizing the MR-Egger’s intercept and its p-value. If this intercept approached zero (<0.1) and the p-value exceeded 0.05, we excluded the effect of pleiotropy at the IV level. For better validation of the potentially abnormal SNPs and the horizontal pleiotropy, the Mendelian random pleiotropic residuals and outliers (MR-PRESSO) approach was incorporated [31].

The eligible SNPs were subjected to sensitivity analyses through the aid of Leave-one-out. This method primarily calculates the MR results of the remaining IVs after excluding IVs one by one, regarded as reliable if unobvious alterations are noted in the overall error bars after the exclusion of every SNP.

### Statistical power

We calculated the statistical power of MR through online statistics (https://shiny.cnsgenomics.com/mRnd/) [32], finding a type I error of 0.05 and K (case composition ratio) values of 54%, 40%, and 17% for all BC, ER+ BC, and ER-BC, respectively. R^2^_xz_ is the variance proportion explainable for the SNP–exposure correlation, whose computational formula is: 2×EAF×(1-EAF)×beta^2^ [33]. Here, EAF and Beta refer separately to the effect of allele frequencies and the predicted influence of genetics during exposure. The R^2^_xz_ for schizophrenia was 20.93%. The sample size for the BC pooled data was 228,951, having 122,977 cases and 105,974 controls. We were sufficiently competent (>80%) when the expected odds ratio (OR) for schizophrenia was≥1.03. Power estimates of BC subtypes for schizophrenia IV are represented in S3 Table in S1 Data.

Data were analyzed in this study through the TwoSampleMR ver. 0.5.6 plus the R ver. 4.1.0. Differences were considered significant when P < 0.05.

## Results

### The two-sample MR estimates for schizophrenia, BD, and MDD with the risk of BC

#### MR estimates for schizophrenia–BC susceptibility association

Based on evidence, the genetically-predicted schizophrenia was associated with all BC (OR = 1.06, 95% CI 1.02–1.10, P = 0.001), ER-BC (OR = 1.06, 95% CI 1.01–1.11, P = 0.0123), and ER+ BC (OR = 1.06, 95% CI 1.03–1.10, P = 0.0009) risks (Table 1). Nine palindromic SNPs were excluded (S4 Table in S1 Data). The F-statistics range for schizophrenia-related SNPs was 29.65–143.41, indicating a powerful IVs–exposure association. With the schizophrenia-related SNPs, 20.93% of the genetic variation was understandable.

**Table 1:** The MR estimate results of between schizophrenia BD and MDD and breast cancer risk.

		All breast cancer			ER+ breast cancer			ER-breast cancer		
Exposure	nsnp	OR (95% CI)	P-value	P-value for pleiotropy or heterogeneity	OR (95% CI)	P-value	P-value for pleiotropy or heterogeneity	OR (95% CI)	P-value	P-value for pleiotropy or heterogeneity
**schizophrenia**										
Inverse variance weighted	68	1.06 (1.02 1.10)	0.001	0.5314	1.06 (1.03 1.10)	0.0009	0.4344	1.06 (1.01.1.11)	0.0123	0.6212
MR-Egger	68	1.10 (0.96 1.26)	0.1564	2.85E-10	1.13 (0.97 1.31)	0.1174	6.85E-07	1.01 (0.85.1.21)	0.8865	0.0818
Weighted median	68	1.03 (1.00 1.07)	0.0554		1.04 (1.00 1.08)	0.044		1.07 (1.01.1.13)	0.0316	
**BD**										
Inverse variance weighted	10	1.05 (0.96 1.14)	0.2809	0.2435	1.03 (0.94 1.12)	0.4964	0.4348	1.00 (0.86.1.15)	0.9578	0.1499
MR-Egger	10	1.45 (0.87 2.42)	0.1922	0.0014	1.29 (0.75 2.22)	0.3842	0.0226	1.89 (0.85.4.20)	0.157	0.0089
Weighted median	10	0.99(0.92 1.07)	0.8832		1.01 (0.93 1.09)	0.8709		0.93(0.82.1.07)	0.3142	
**MDD**										
Inverse variance weighted	39	1.08 (0.97 1.20)	0.1436	0.4538	1.03 (0.91 1.17)	0.6453	0.2511	1.05 (0.90.1.21)	0.5361	0.9468
MR-Egger	39	1.35 (0.75 2.42)	0.3188	0.0002	1.56 (0.77 3.15)	0.2263	0.0001	1.08 (0.47.2.44)	0.8604	0.2191
Weighted median	39	1.08(0.97 1.21)	0.1642		0.99 (0.87 1.14)	0.9135		1.00(0.81.1.22)	0.9628	

BD bipolar disorder, MDD major depressive disorder, MR Mendelian randomization, nsnp number of single nucleotide polymorphism, OR odds ratio, CI confidence interval.

P-value for pleiotropy based on MR-Egger intercept.

P-value for heterogeneity based on Q statistic.

The estimates were derived from a random effects model due to the P-value<0.05 for heterogeneity based on Cochran’s Q statistic, otherwise, The estimates were derived from a fixed-effects MR estimates model.

#### MR estimates for BD–BC risk association

The causal association of BD with all BC risk was supported by little evidence (OR = 1.05, 95% CI 0.96–1.14, P = 0.2809) (Table 1). The association with ER-BC (OR = 1.00, 95% CI 0.86–1.15, P = 0.9578) and ER+ BC (OR 1.03, 95% CI 0.94–1.12, P = 0.4964) were similar. As displayed in S4 Table in S1 Data, we eliminated five palindromic SNPs. Within an F-statistics range of 29.66–56.81, the BD-related SNPs were associated significantly with the exposure. It was observed that 4.92% of the overall genetic variation was explainable through the BD-related SNPs.

#### MR estimates for MDD–BC risk association

MR estimates showed that the genetically-predicted MDD was insignificantly associated with all BC (OR = 1.08, 95% CI 0.97–1.20, P = 0.1436) (Table 1). As revealed by the subgroup analysis, less evidence supported the causal correlations of MDD with the ER-BC (OR = 1.05, 95% CI 0.90–1.21, P = 0.5361) and the ER+ BC (OR = 1.03, 95% CI 0.91–1.17, P = 0.6453) risks. Additionally, nine palindromic SNPs were excluded (S4 Table in S1 Data). The F-statistics range for MDD-related SNP was 30–78.45, implying that the weak instrumental bias barely affects the SNP. It was observed that 1.78% of the overall genetic variation was explainable through the MDD-related SNPs. S1–S6 Figs shows the scatter and forest plots of schizophrenia, BD, MDD, and BC.

#### The two-step MR estimates for mediators-associated SNPs between schizophrenia and BC risk

A two-step MR study explored the potential role of mediators through the effect sizes of schizophrenia to the mediators (Table 2), in which schizophrenia and smoking had a significant correlation (beta1 = 0.0438, P = 0.0058). In a multivariate MR analysis of mediators to outcomes, smoking, alcohol consumption, and BMI were associated with the occurrence of BC. Among them, smoking revealed a significant correlation with all BC (beta2 = 0.1467, P = 0.0166). Significant connections of alcohol intake to all BC and ER-BC were noted. Besides its significant linkage to all BC, BMI was also found to be significantly linked to both ER-BC and ER+BC in the subgroup analysis (Table 3). Beta0, beta1, and beta2 were all significant, indicating a causal relationship between exposure and outcome, and mediator variables could partly mediate this relationship. Therefore, in the causal relationship analysis of smoking in schizophrenia and BC risk, beta0, beta1, and beta2 were all significant. It depicted that smoking could mediate schizophrenia leading to an elevated all BC risk, revealing a final causal relationship of 11.29% mediated through smoking.

**Table 2:** The MR effect of schizophrenia on mediators.

							heterogeneity test		pleiotropy test		
Exposure	outcomes	Method	N of SNPs	Beta	SE	P-value	Q statistic	Q p-value	Egger intercept	SE	P-value
Schizophrenia	smoking initiation	MR-Egger	66	0.0155	0.0596	0.7952	445.7729	1.39E-58	0.0024	0.0049	0.6250
		Weighted median	66	0.0333	0.0115	0.0038					
		Inverse variance weighted	66	0.0438	0.0159	0.0058	447.4533	1.79E-58			
Schizophrenia	Alcohol consumption	MR-Egger	68	-0.0673	0.1557	0.6668	75.7421	0.1930	0.0094	0.0123	0.4471
		Weighted median	68	0.0307	0.0433	0.4781					
		Inverse variance weighted	68	0.0492	0.0322	0.1263	76.4135	0.2019			
Schizophrenia	Physical activity	MR-Egger	67	0.0220	0.0223	0.3284	171.6380	1.46E-11	-0.0009	0.0018	0.6234
		Weighted median	67	0.0116	0.0059	0.0497					
		Inverse variance weighted	67	0.0114	0.0060	0.0586	172.2809	1.96E-11			
Schizophrenia	type 2 diabetes	MR-Egger	58	0.0013	0.0014	0.3837	58.5913	0.3806	-0.0001	0.0001	0.3737
		Weighted median	58	0.0001	0.0004	0.8210					
		Inverse variance weighted	58	3.31E-06	0.0003	0.9914	59.4325	0.3871			
Schizophrenia	Prolactin levels	MR-Egger	72	0.0863	0.1156	0.4576	78.9392	0.2173	-0.0055	0.0093	0.5587
		Weighted median	72	0.0238	0.0389	0.5399					
		Inverse variance weighted	72	0.0204	0.0277	0.4613	79.3286	0.2331			
Schizophrenia	Body mass index (BMI)	MR-Egger	67	0.0529	0.0414	0.2053	736.6295	1.79E-114	-0.0060	0.0034	0.0825
		Weighted median	67	-0.0232	0.0068	0.0007					
		Inverse variance weighted	67	-0.0173	0.0114	0.1292	771.8701	5.97E-121			

MR Mendelian randomization,SE standard deviation.

The estimates were derived from a random effects model due to the P-value<0.05 for heterogeneity based on Cochran’s Q statistic, otherwise,The estimates were derived from a fixed-effects MR estimates model.

**Table 3:** The MR effect of mediators on BC.

	All BC			ER+ BC			ER- BC		
exposure	Beta	SE	P-value	Beta	SE	P-value	Beta	SE	P-value
smoking initiation	0.1467	0.0612	0.0166	0.1219	0.0666	0.0673	0.0826	0.0896	0.3566
Alcohol consumption	-0.0620	0.0288	0.0312	0.0212	0.0370	0.5660	-0.0984	0.0421	0.0193
Physical activity	-0.2570	0.1475	0.0814	-0.1255	0.1625	0.4400	-0.2241	0.2162	0.2998
type 2 diabetes	1.8138	2.5483	0.4766	0.1219	0.0666	0.0673	2.5023	3.7323	0.5026
Prolactin levels	0.0155	0.0336	0.6452	0.0212	0.0370	0.5660	0.0007	0.0492	0.9880
Body mass index (BMI)	-0.1992	0.0433	4.24E-06	-0.1810	0.0477	0.0001	-0.1968	0.0635	0.0019

MR Mendelian randomization,SE standard deviation,BC breast cancer.

### Sensitivity analyses

According to the heterogeneity test results, several significant differences were noted in the estimated causal associations of exposures with all BC risks (S5 Table in S1 Data). Thus, these models were applied with random-effects MR estimates when the Q p-value<0.05; otherwise, fixed-effects MR estimates were used. Based on the horizontal pleiotropy assessment by MR-Egger intercept and the p-values, such pleiotropy did not affect the MR estimates for exposures and BC (S6 Table in S1 Data).

**Fig 1:**
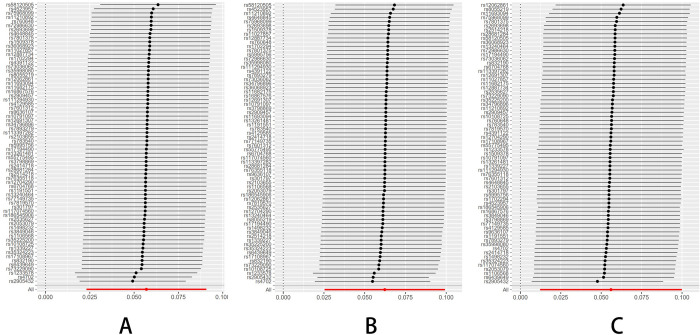
MR Leave-one-out plot for schizophrenia on breast cancer. (A) All BC. (B) ER+ BC. (C) ER-BC.

**Fig 2:**
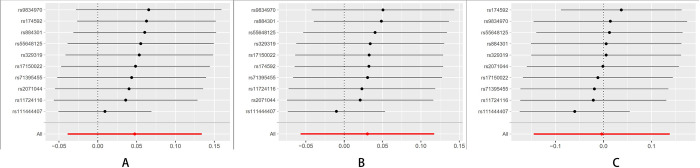
MR Leave-one-out plot for BD on breast cancer. (A) All BC. (B) ER+ BC. (C) ER-BC.

**Fig 3:**
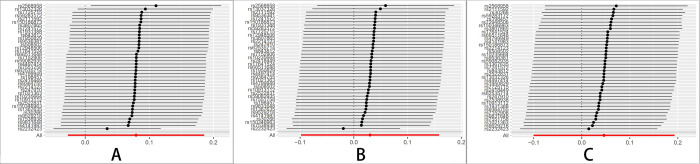
MR Leave-one-out plot for MDD on breast cancer. (A) All BC. (B) ER+ BC. (C) ER-BC.

The Leave-one-out sensitivity test revealed that the result alterations were unobvious no matter which schizophrenia, BD, and MDD-related SNP was eliminated, indicating the high robustness of the MR results (Figs 1–3).

No potential pleiotropy was detected in the MR estimation of exposures–BC risk casualty by the MR-PRESSO approach, despite discovering several outliers (S7 Table in S1 Data). For all BC and ER+ BC, after the SNPs removal in restrictive MR analysis, schizophrenia was found to be significantly associated with the risks of ER+ BC (OR 1.05, 95% CI 1.02–1.09, P = 0.0009) and all BC (OR 1.05, 95% CI 1.02–1.08, P = 0.0003). Outlier SNPs were not identified after applying the MR-PRESSO method in ER-BC. No potential pleiotropy was detected in the MR estimation during causal correlations of BD and MDD with BC risk by the MR-PRESSO approach, and it found little cause relationship. The scatter, forest, and Leave-one-out plots depending on MR-PRESSO applied after excluding outlier SNPs for exposures on BC are shown in S7–S15 Figs.

## Discussion

As indicated by the present TSMR analysis covering over 122,977 BC patients and 105,974 healthy controls, BC susceptibility is influenced etiologically by schizophrenia. The schizophrenic population is more susceptible to BC, irrespective of the estrogen levels. As revealed by the sensitivity analyses, outcomes are influenced through IVs by exposure only, instead of pathways like confounding ones, suggestive of the absence of pleiotropy.

The latest global burden of disease data indicates a significant increase in the raw prevalence, incidence, and burden of schizophrenia between 1990 and 2019 [34].The latest meta-analysis [35] depicted that the schizophrenic population is more susceptible to BC, but there is significant heterogeneity among the included studies, which may be due to confounding factors. Mendelian randomization (MR) research, unlike clinical studies, is less susceptible to clinical confounding factors. We assessed the schizophrenia–BC casualty based on a two-sample and two-step MR strategy, finding that BC was more prevalent among the schizophrenic population, consistent with previous MR studies [36, 37]. Some behaviors in patients with schizophrenia are risk factors for BC, including obesity, childlessness, and use of anti-schizophrenia drugs leading to elevated prolactin levels. We also performed a two-step MR analysis on confounding factors and found that smoking explained 11.29% of the causal relationship between schizophrenia and BC risk. Furthermore, accumulating evidence that the underlying mechanisms of schizophrenia and BC share a common genetic basis [38]. Studies have shown that [13] anti-schizophrenia drug use for more than 5 years is associated with BC risk but this association was not found in our study, which is limited by the selected database. BC patients with schizophrenia have a two-fold increase in the all-cause mortality hazard [9]. Patients with schizophrenia have less BC screening than the general population [6, 39, 40], and poor treatment adherence [3, 10], which may have contributed to higher mortality in BC patients with schizophrenia. It suggests that we should strengthen the screening and prevention of BC in patients with schizophrenia.

Bipolar disorder refers to a range of psychiatric illnesses with both manic or hypomanic episodes and depressive episodes that are recurrent, chronic, and severe. Several researchers have now focused on its association with the incidence of carcinomas. The causality of BD and BC risk is controversial [41, 42]. In our MR research, BD failed to be linked to BC incidence, and our results differ from the published studies [43]. Although more IVs for exposure-related SNPs were used in this article, the IVs used for exposure-related in this article included Asian populations [43]. In contrast, the outcome-related IVs were from the European populations, leading to errors in the results [43].

Major depressive disorder (MDD) is a common illness among women where the incidence is twice as high as in men. The 1-year prevalence of MDD is approximately 6% [44]. Previous MR studies [45] have shown that MDD increases the risk of developing BC, whereas our study did not observe a similar result. In our study, the linkage disequilibrium (LD) was removed according to kb = 10000, r2<0.001 where there is less confounding and pleiotropy while the previous study set kb = 3000, r2<0.1. Further, numerous studies have shown that MDD is more common after BC diagnosis and emphasize the importance of MDD in BC prognosis [46, 47]. Few works of literature have investigated the relationship between MDD and the BC occurrence, and clinical studies have not shown a positive association [48, 49]. Dysthymia with major depression is defined as having MDD during periods of dysthymia [50]. It is speculated that persistent depression status and intensity of depression are additional risk factors in the development of BC, and the duration and intensity of depression are more critical. It is necessary for the stratified analysis of MDD and BC risk. Unfortunately, the data of MDD-related SNP we obtained cannot be stratified.

Our study’s strength lies in identifying a causal relationship between schizophrenia and the incidence of BC using the MR method. Additionally, we discovered that smoking plays a certain mediating role in this relationship. The results indicated that smoking mediates schizophrenia to elevate the risk of BC, suggesting we should improve BC screening for mentally ill patients and manage unhealthy behaviors, especially smoking.

Likewise, our study has some limitations. First, other confounding factors, those not available (eg age stratification of BC populations and duration and intensity of schizophrenia exposure) may also have influenced the results. Second, mediating exposure factors only explore the common influencing factors. Third, because this study relied on pooled data from GWAS of European ancestry, the generalization of this result should be validated in other ethnic groups.

In future research, larger-scale clinical studies should be conducted to comprehensively assess the relationship between schizophrenia and the incidence risk of BC. Additionally, clinicians should delve deeper into exploring the underlying mechanisms linking schizophrenia and BC to facilitate the development of more effective intervention strategies.

In terms of clinical application, BC risk should be integrated into the healthcare management plans of individuals with schizophrenia. This could include measures such as health education, smoking cessation support, and regular BC screening. Furthermore, psychiatrists and oncologists should actively engage in interdisciplinary collaboration to better manage the co-occurrence of these distinct diseases across different medical domains.

## Conclusions

In conclusion, our study utilizing the two-sample Mendelian randomization method underscores the significance of schizophrenia as a potential risk factor for BC. Smoking was identified as a mediating factor in this relationship. These findings highlight the need for enhanced health management strategies for individuals with schizophrenia. Further exploration of biological mechanisms and interdisciplinary collaboration will guide future interventions and public health policies in mitigating BC risk in this vulnerable population.

## Supporting information

S1 Fig MR forest plot for schizophrenia on breast cancer. (A) All BC. (B) ER+ BC. (C) ER-BC.
(PDF)

S2 Fig MR scatter plot for schizophrenia on breast cancer. (A) All BC. (B) ER+ BC. (C) ER-BC.
(PDF)

S3 Fig MR forest plot for BD on breast cancer. (A) All BC. (B) ER+ BC. (C) ER-BC.
(PDF)

S4 Fig MR scatter plot for BD on breast cancer. (A) All BC. (B) ER+ BC. (C) ER-BC.
(PDF)

S5 Fig MR forest plot for MDD on breast cancer. (A) All BC. (B) ER+ BC. (C) ER-BC.
(PDF)

S6 Fig MR scatter plot for MDD on breast cancer. (A) All BC. (B) ER+ BC. (C) ER-BC.
(PDF)

S7 Fig MR Leave−one−out sensitivity analysis from MR-PRESSO applied after excluding genetic variants for schizophrenia on breast cancer. (A) All BC. (B) ER+ BC.
(PDF)

S8 Fig MR scatter plot from MR-PRESSO applied after excluding genetic variants for schizophrenia on Breast cancer. (A) All BC. (B) ER+ BC.
(PDF)

S9 Fig MR forest plot from MR-PRESSO applied after excluding genetic variants for schizophrenia on Breast cancer. (A) All BC. (B) ER+ BC.
(PDF)

S10 Fig MR Leave-one-out plot from MR-PRESSO applied after excluding genetic variants for BD on Breast cancer. (A) All BC. (B) ER- BC.
(PDF)

S11 Fig MR scatter plot from MR-PRESSO applied after excluding genetic variants for BD on breast cancer. (A) All BC. (B) ER- BC.
(PDF)

S12 Fig MR forest plot from MR-PRESSO applied after excluding genetic variants for BD on breast cancer. (A) All BC. (B) ER- BC.
(PDF)

S13 Fig MR Leave-one-out plot from MR-PRESSO applied after excluding genetic variants for MDD on breast cancer. (A) All BC. (B) ER+ BC.
(PDF)

S14 Fig MR scatter plot from MR-PRESSO applied after excluding genetic variants for MDD on breast cancer. (A) All BC. (B) ER+ BC.
(PDF)

S15 Fig MR forest plot from MR-PRESSO applied after excluding genetic variants for MDD on breast cancer. (A) All BC. (B) ER+ BC.
(PDF)

S1 Data (XLSX)
