# Supplementary figures and images for: Severe mental illness and the risk of breast cancer: A two-sample, two-step multivariable Mendelian randomization study
Source: PLoS One. 2023 Sep 1;18(9):e0291006. doi: 10.1371/journal.pone.0291006 (PMC10473543; doi:10.1371/journal.pone.0291006)

MR Test

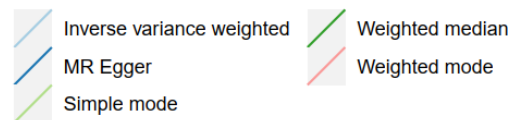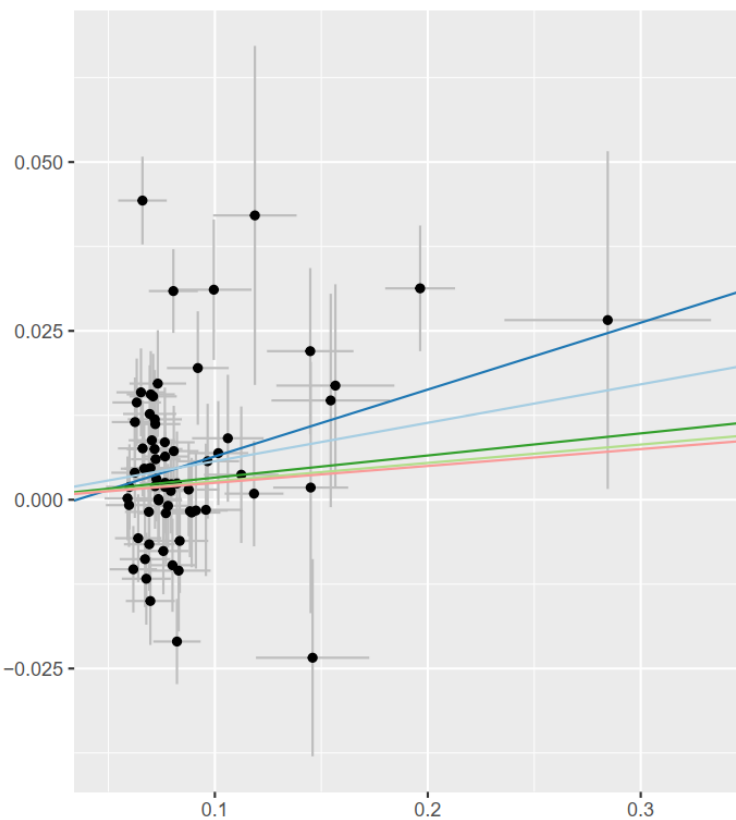

A  
C

MR Test

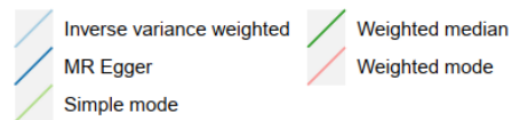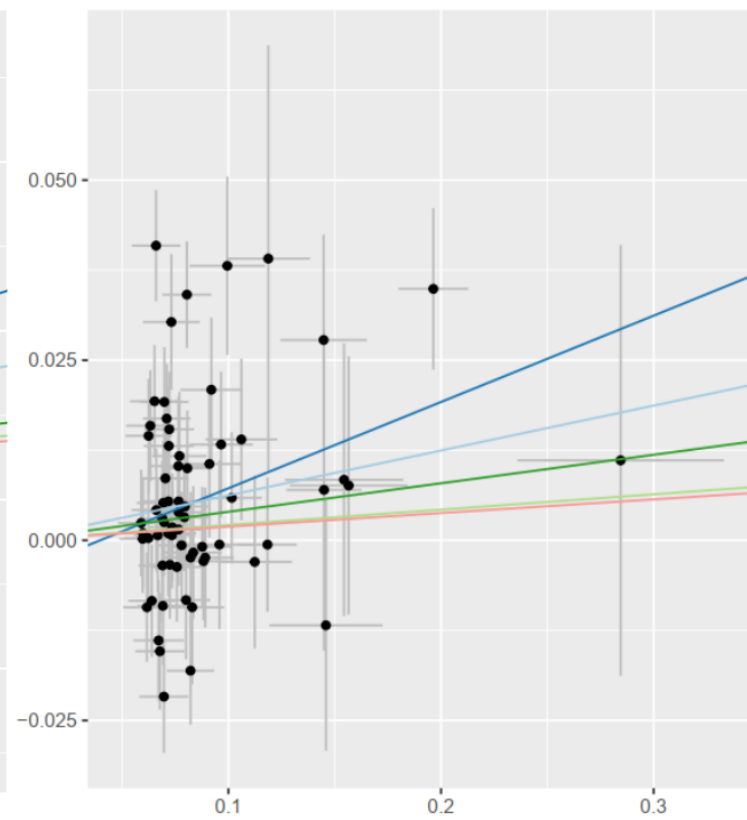

B

MR Test

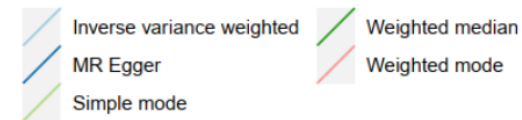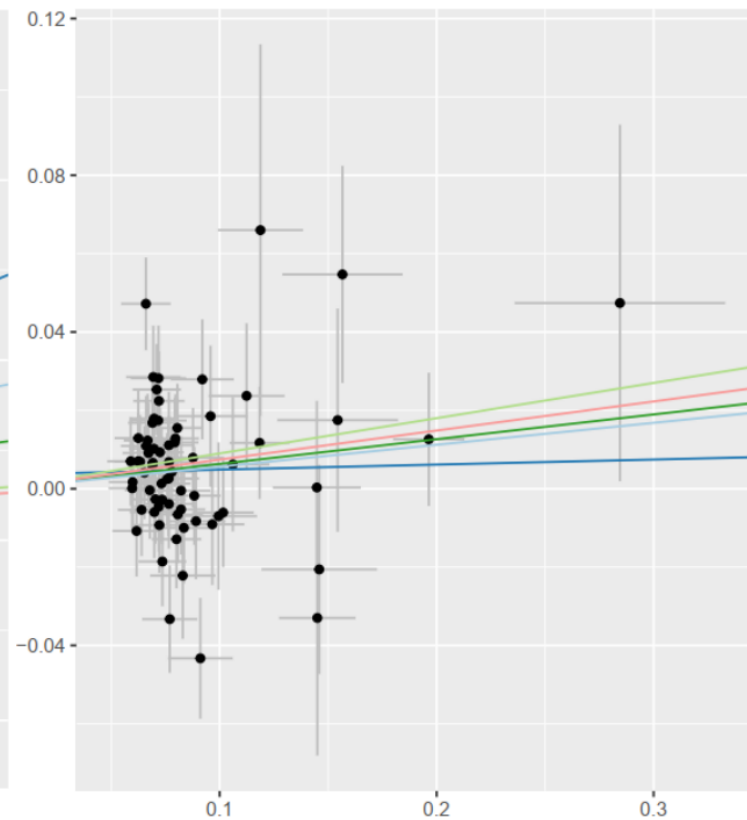

Supplement: S2 Fig — (A) All BC. (B) ER+ BC. (C) ER-BC. (PDF) [file pone.0291006.s002.pdf]

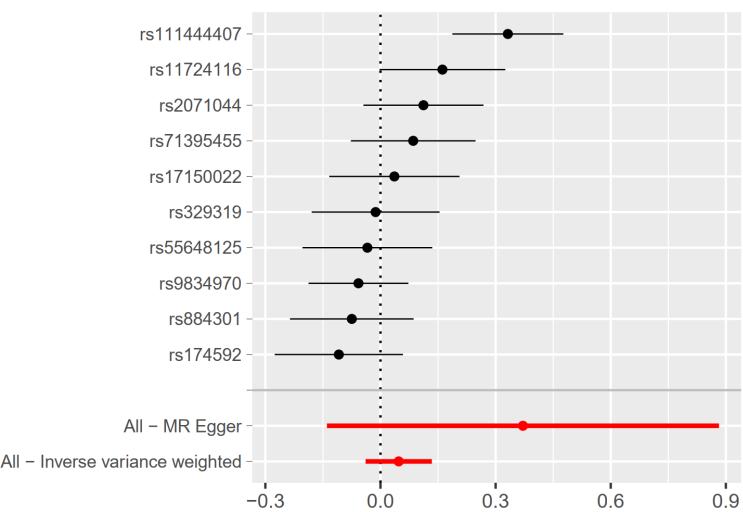

**A**  
**C**

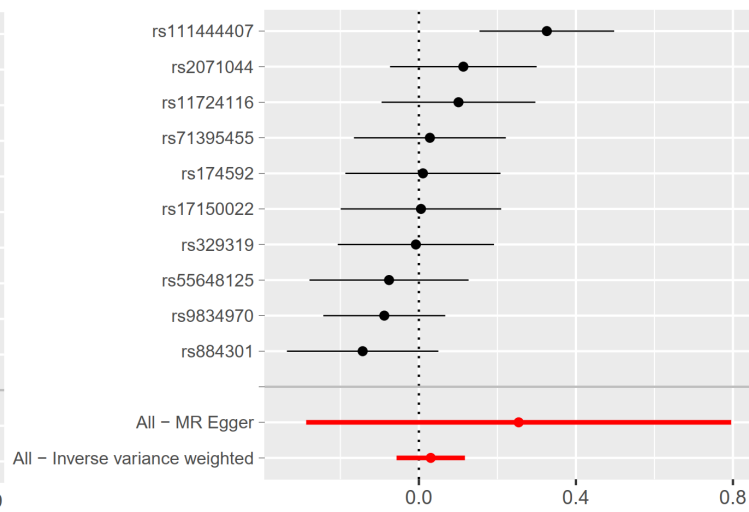

**B**

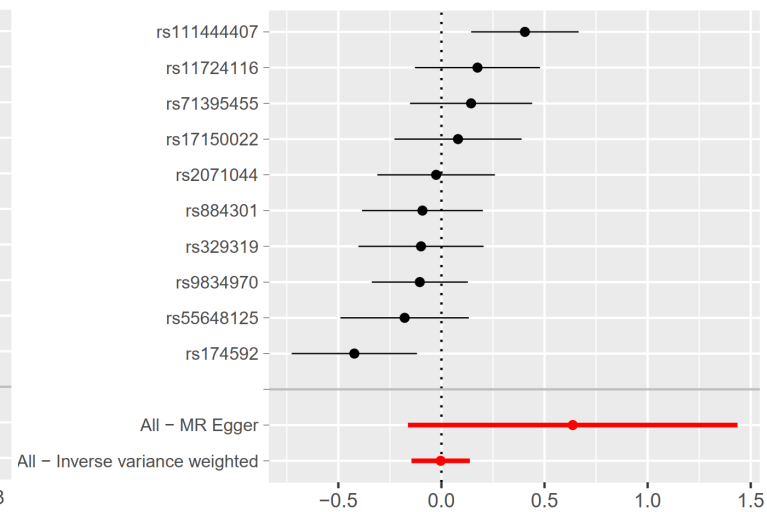

Supplement: S3 Fig — (A) All BC. (B) ER+ BC. (C) ER-BC. (PDF) [file pone.0291006.s003.pdf]

### MR Test

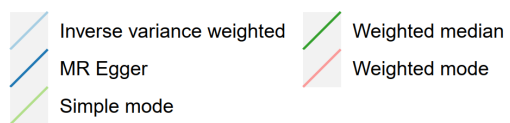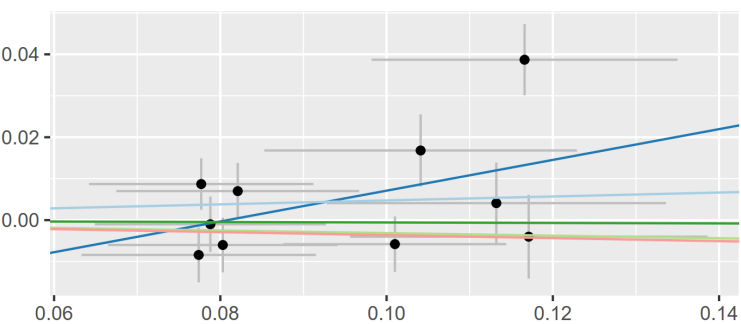

**A**  
**C**

### MR Test

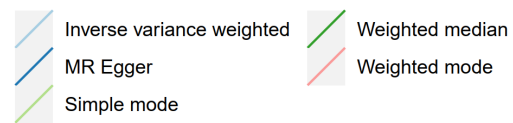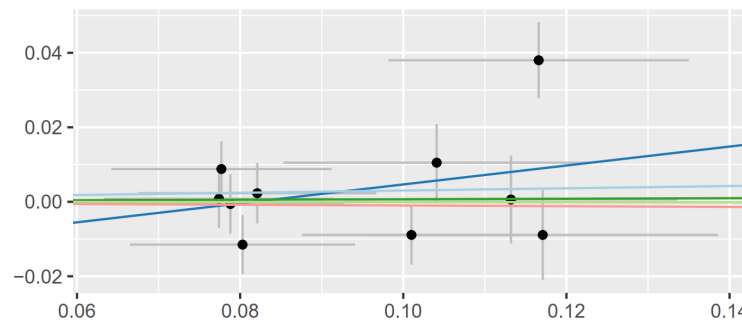

**B**

### MR Test

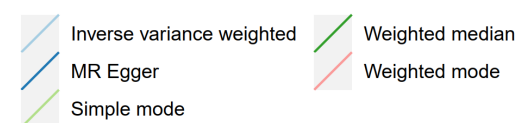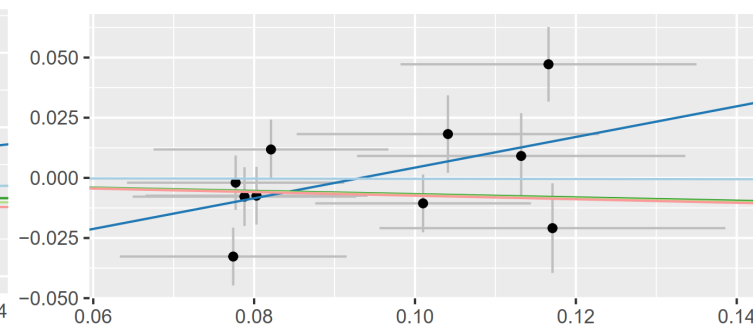

Supplement: S4 Fig — (A) All BC. (B) ER+ BC. (C) ER-BC. (PDF) [file pone.0291006.s004.pdf]

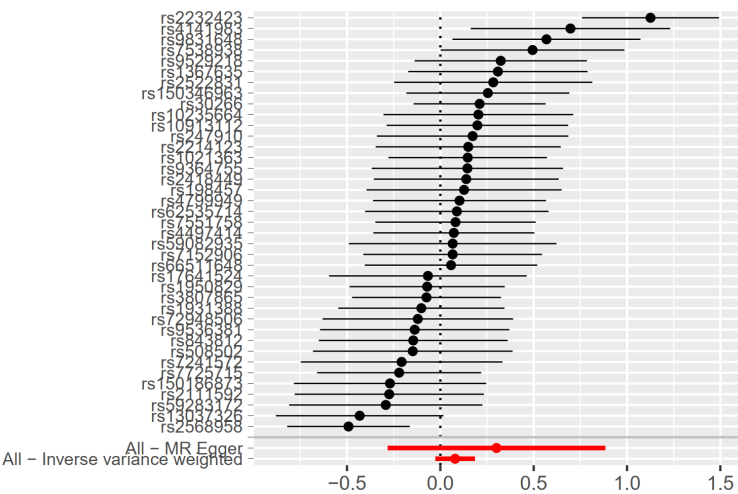

**A**  
**C**

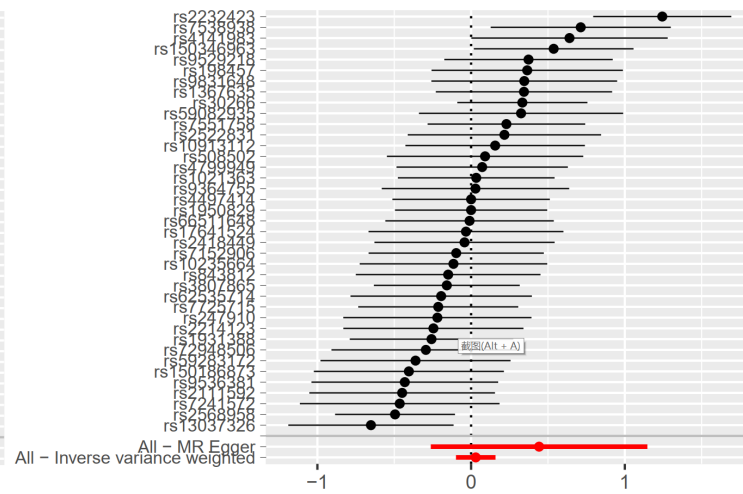

**B**

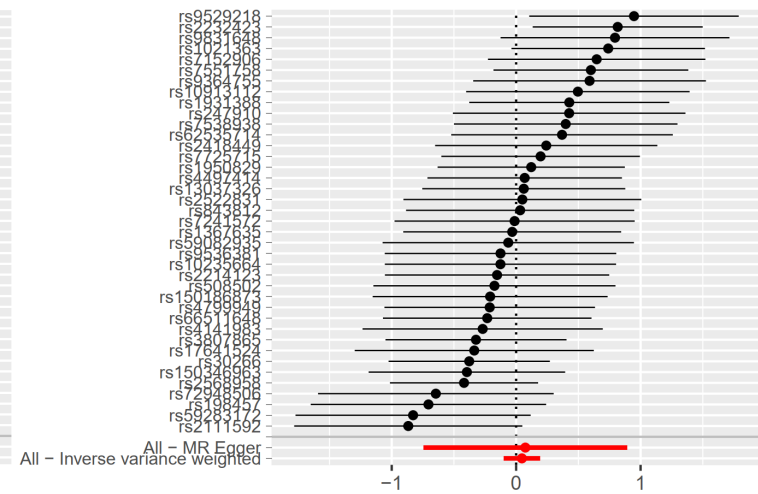

Supplement: S5 Fig — (A) All BC. (B) ER+ BC. (C) ER-BC. (PDF) [file pone.0291006.s005.pdf]

MR Test

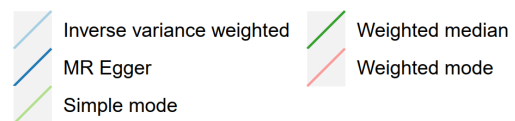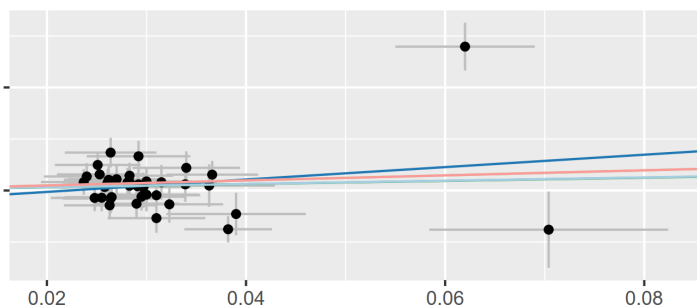

A  
C

MR Test

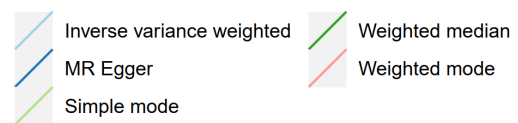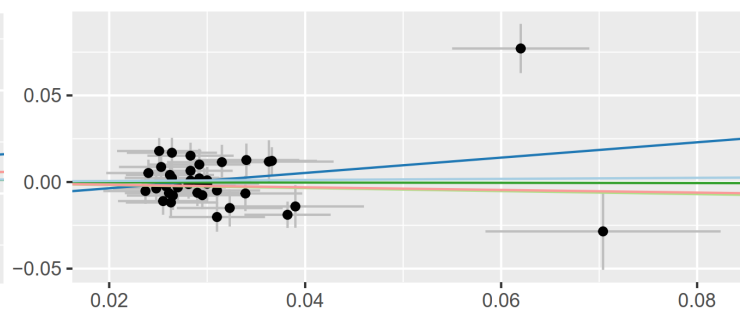

B

MR Test

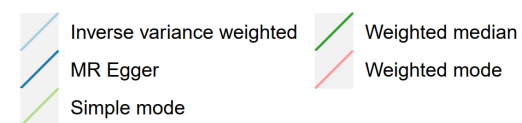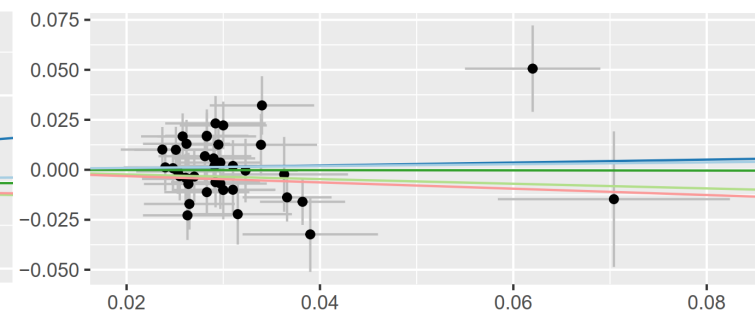

Supplement: S6 Fig — (A) All BC. (B) ER+ BC. (C) ER-BC. (PDF) [file pone.0291006.s006.pdf]

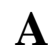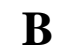

Supplement: S7 Fig — (A) All BC. (B) ER+ BC. (PDF) [file pone.0291006.s007.pdf]

### MR Test

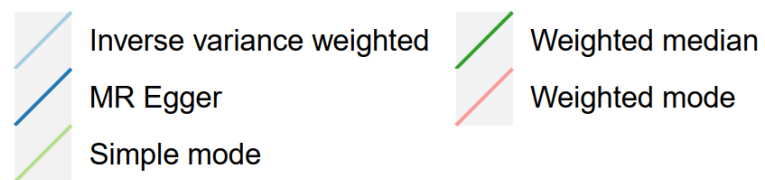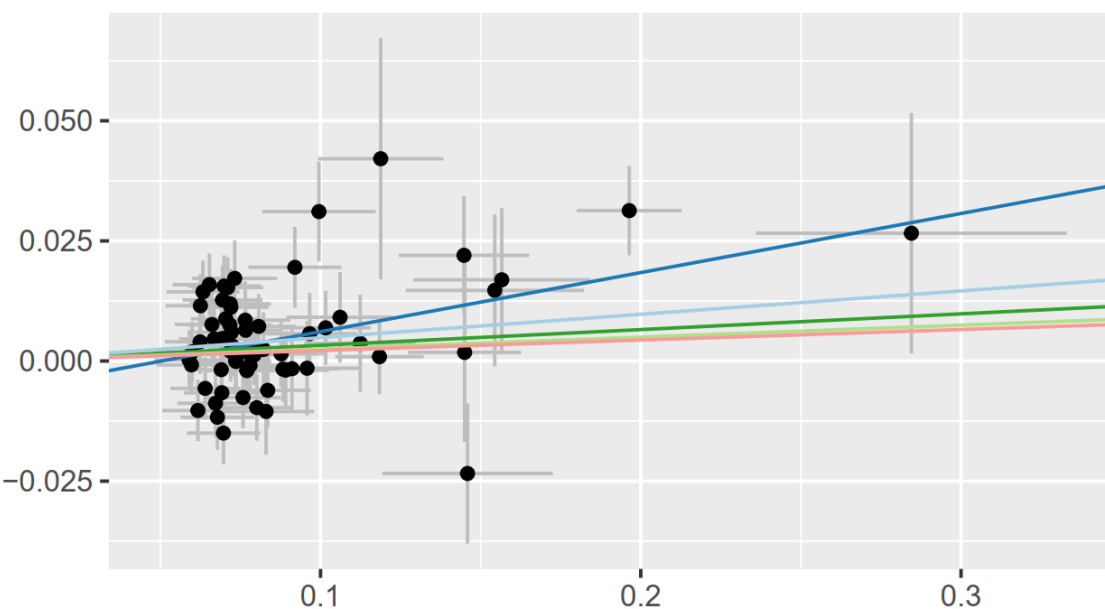

**A**

### MR Test

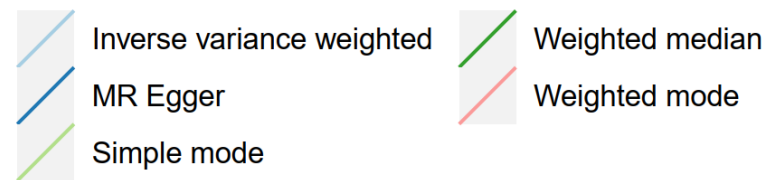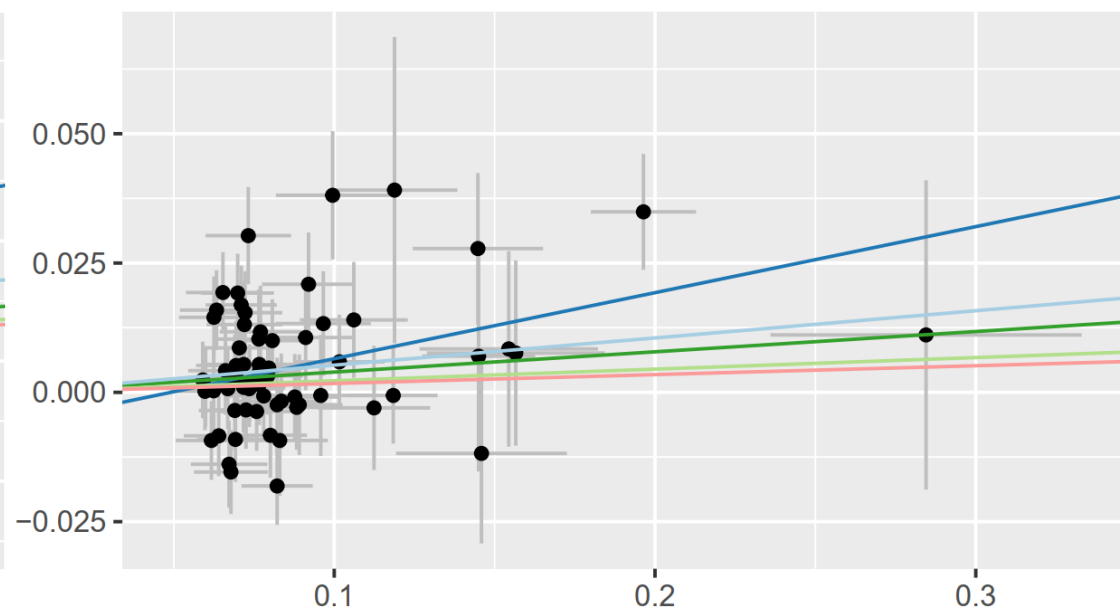

**B**

Supplement: S8 Fig — (A) All BC. (B) ER+ BC. (PDF) [file pone.0291006.s008.pdf]

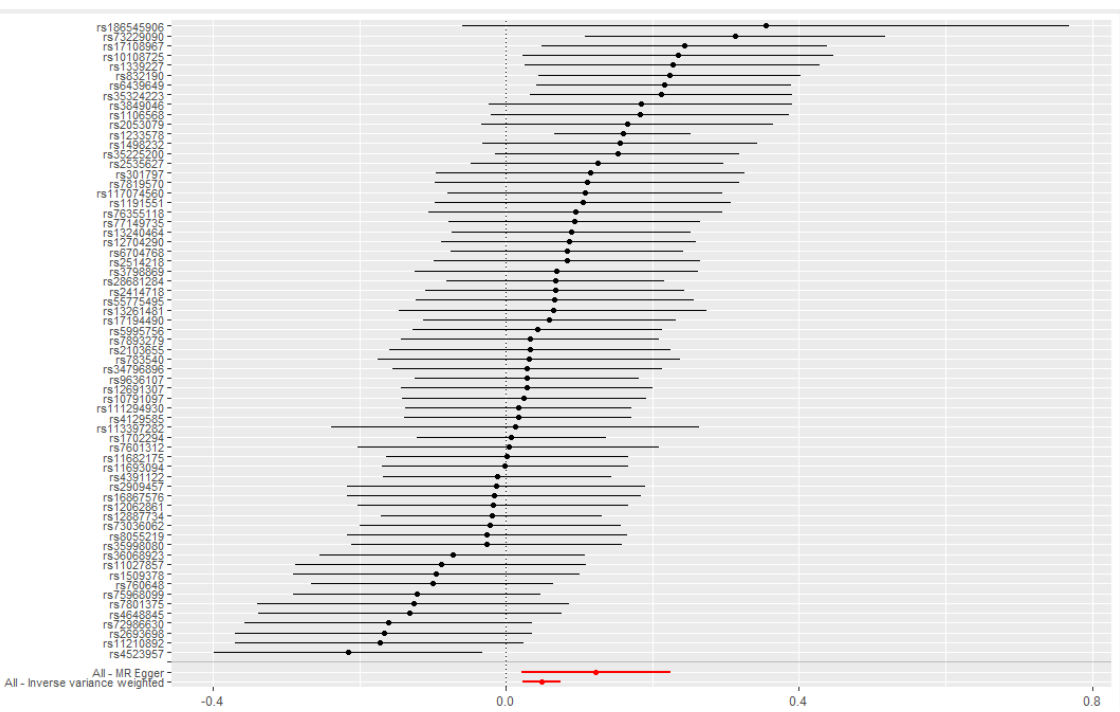

A

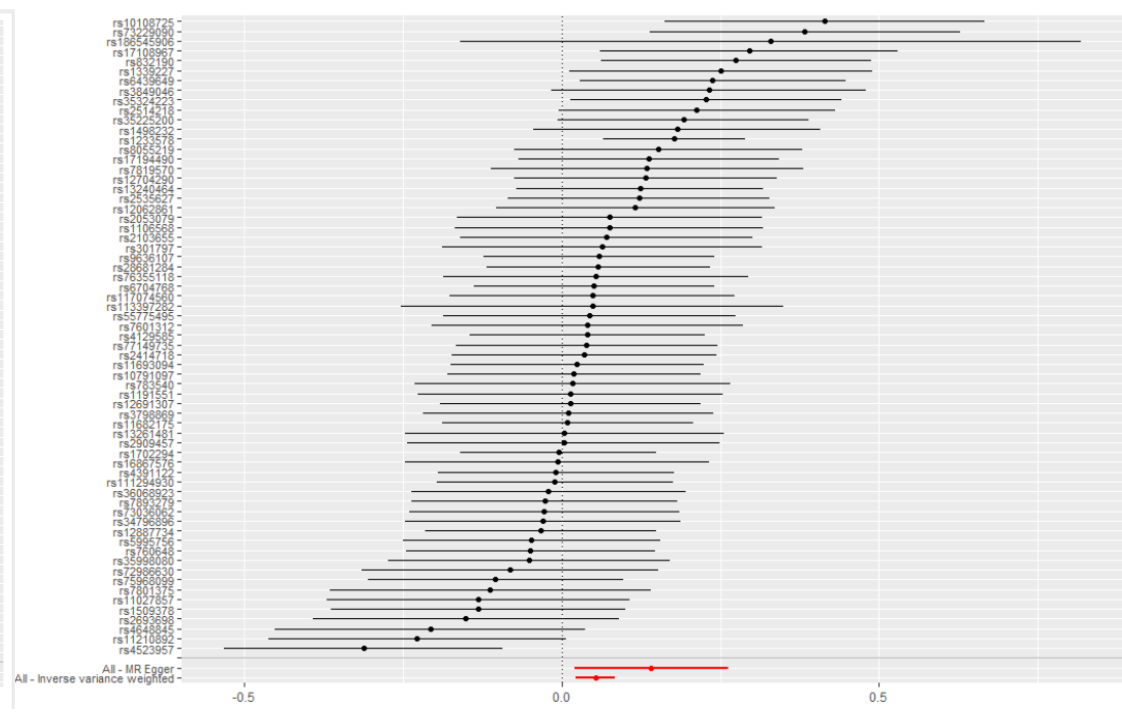

B

Supplement: S9 Fig — (A) All BC. (B) ER+ BC. (PDF) [file pone.0291006.s009.pdf]

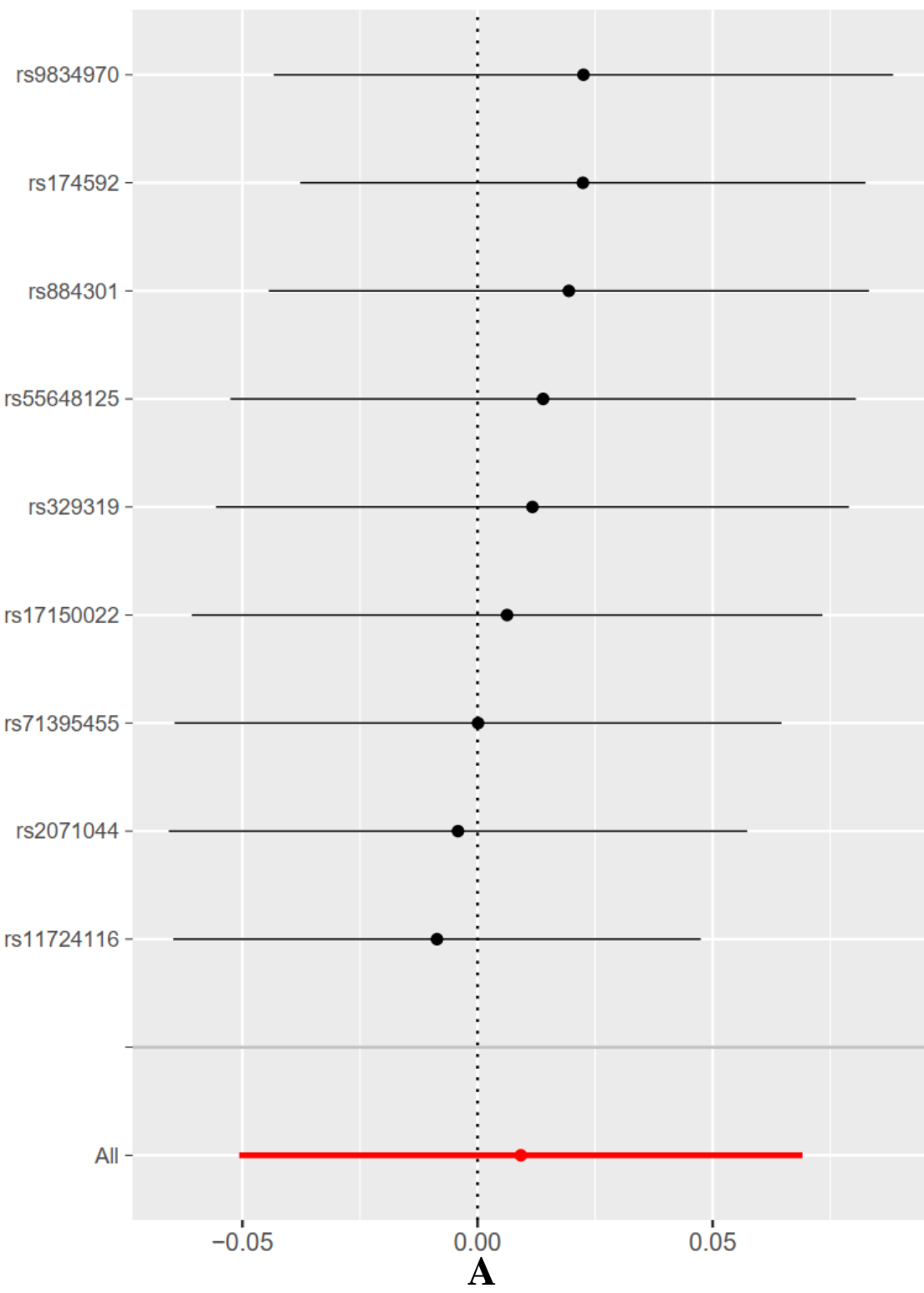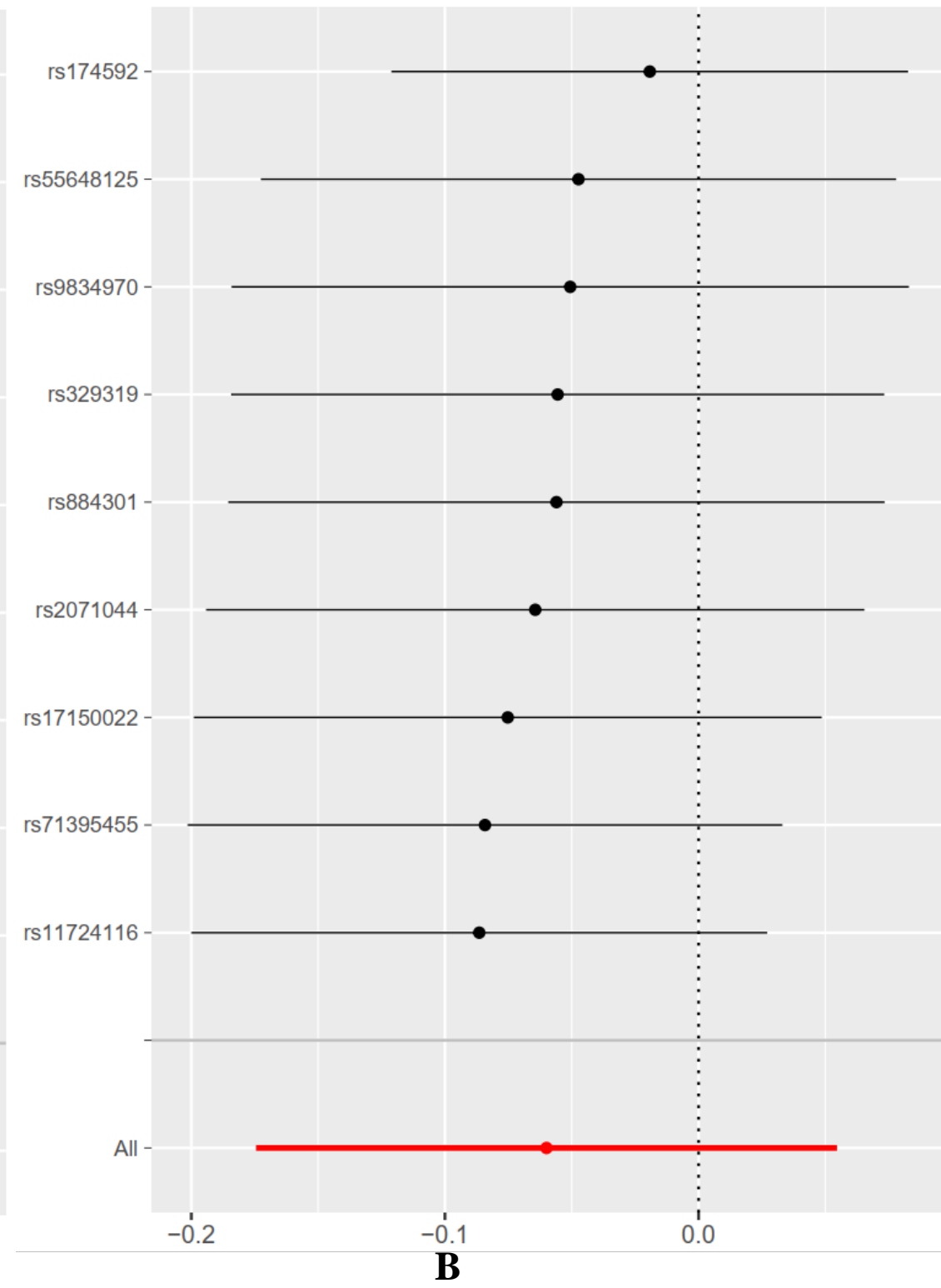

Supplement: S10 Fig — (A) All BC. (B) ER- BC. (PDF) [file pone.0291006.s010.pdf]

# MR Test

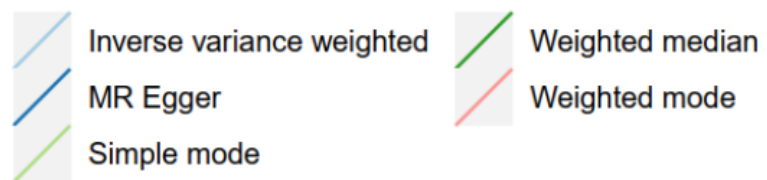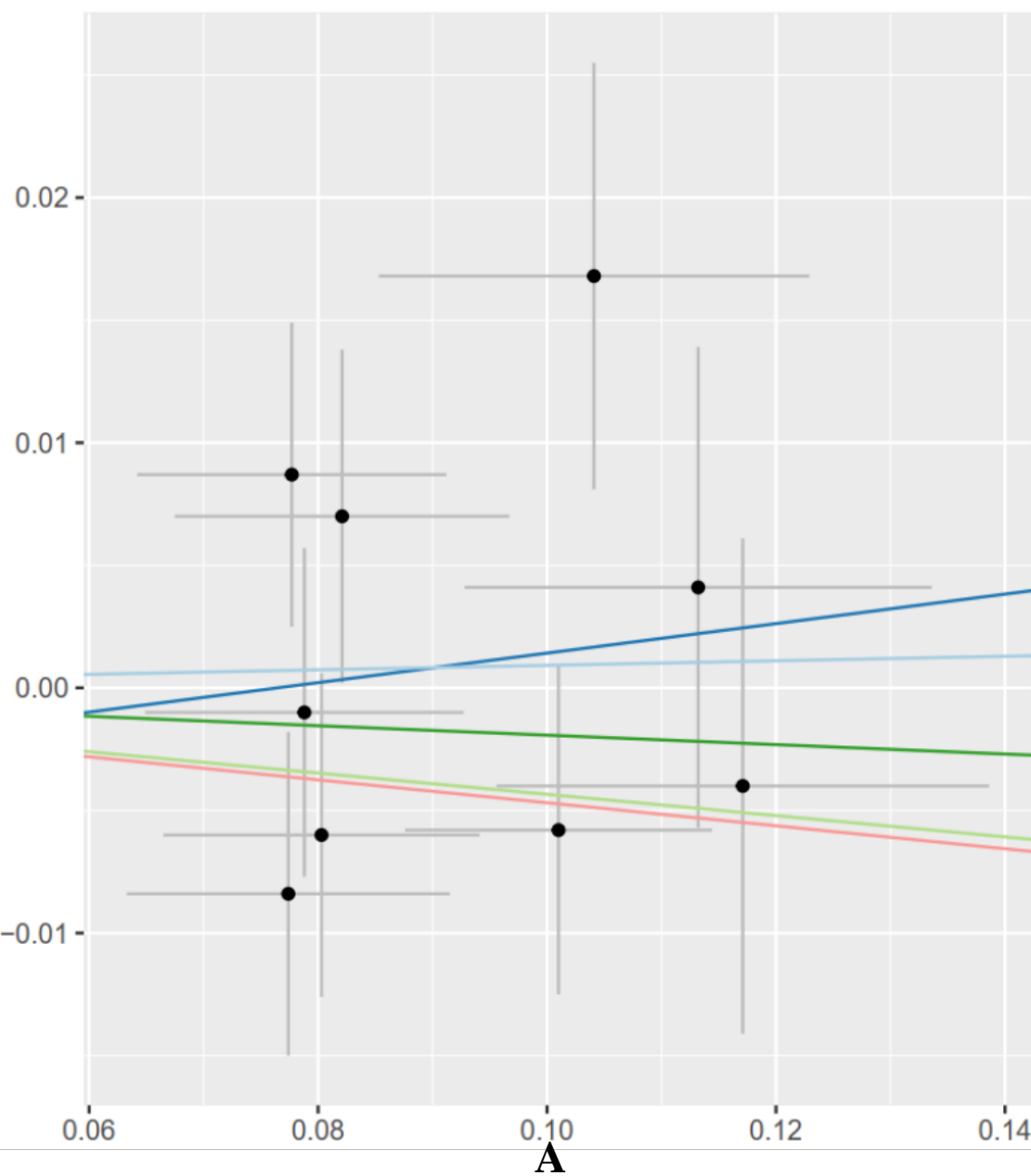

# MR Test

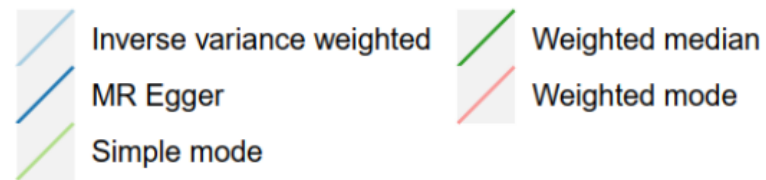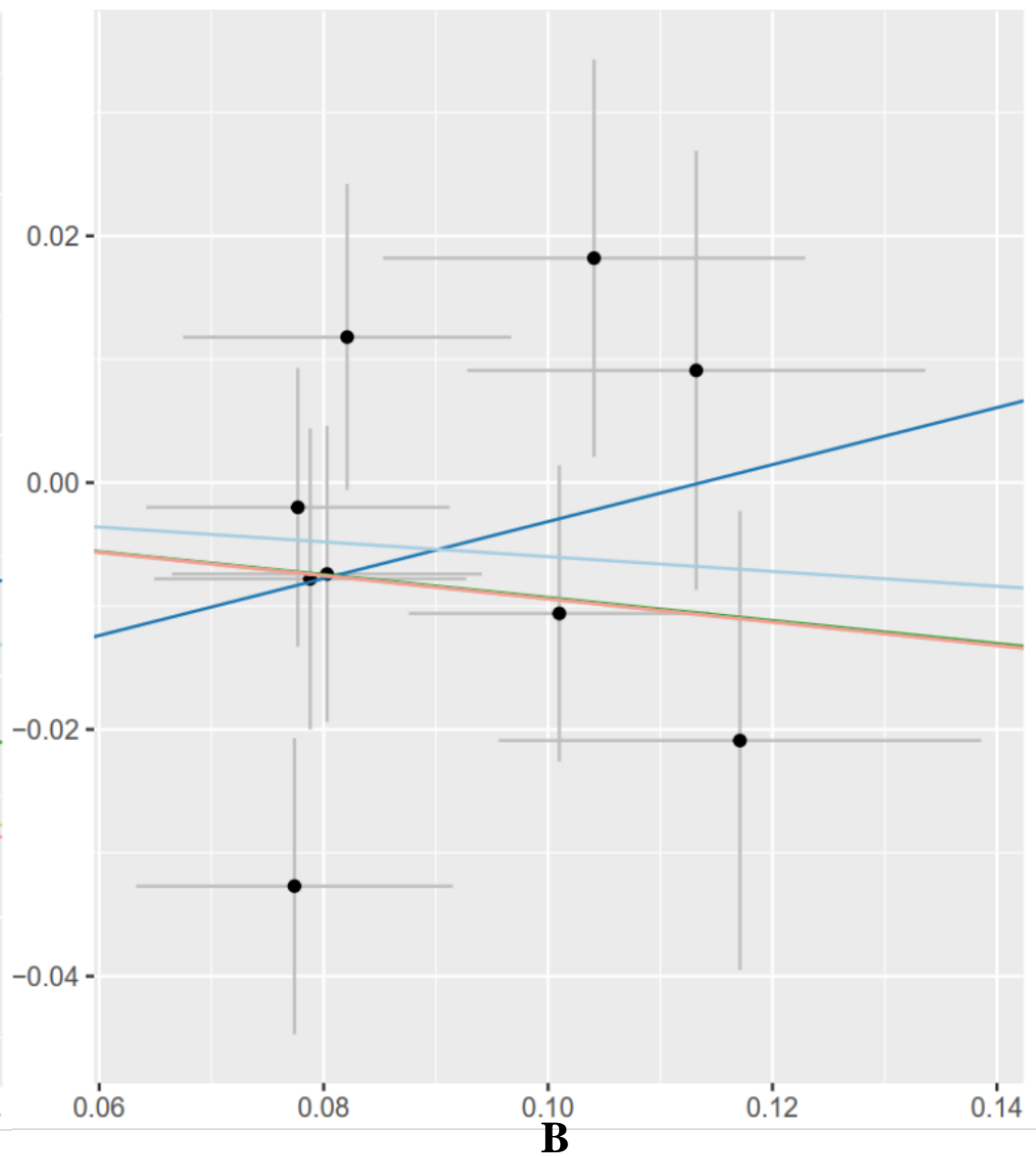

Supplement: S11 Fig — (A) All BC. (B) ER- BC. (PDF) [file pone.0291006.s011.pdf]

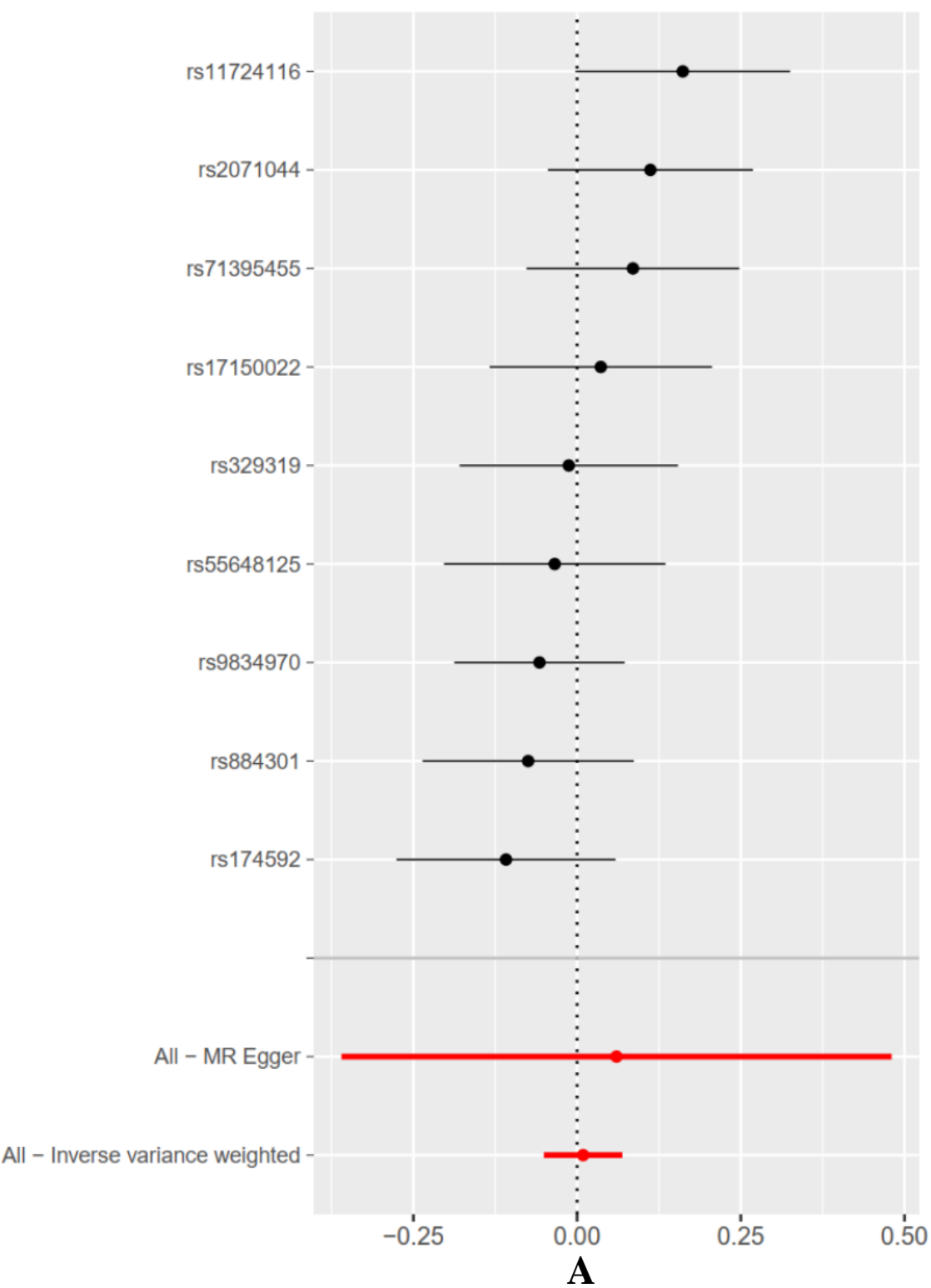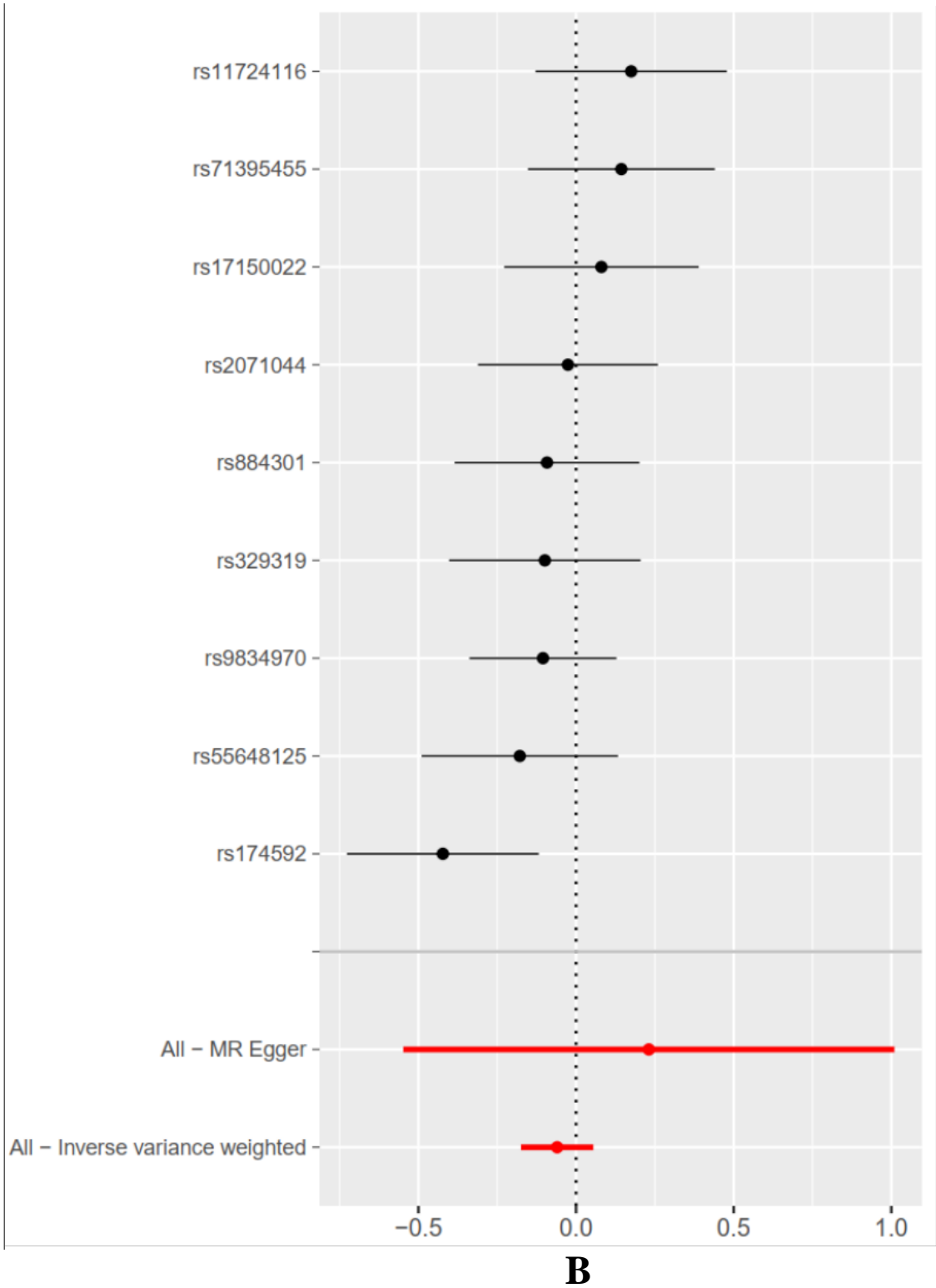

Supplement: S12 Fig — (A) All BC. (B) ER- BC. (PDF) [file pone.0291006.s012.pdf]

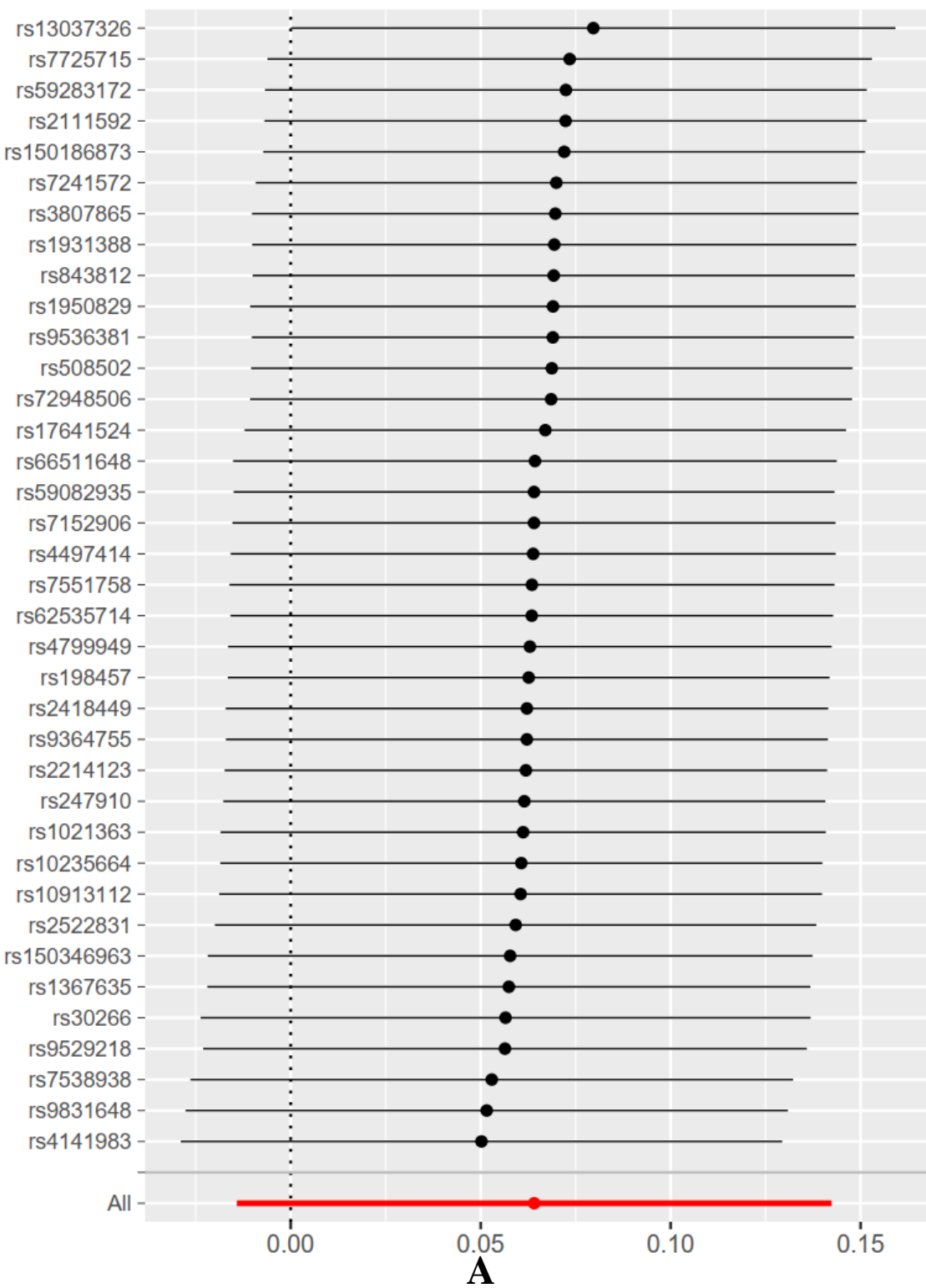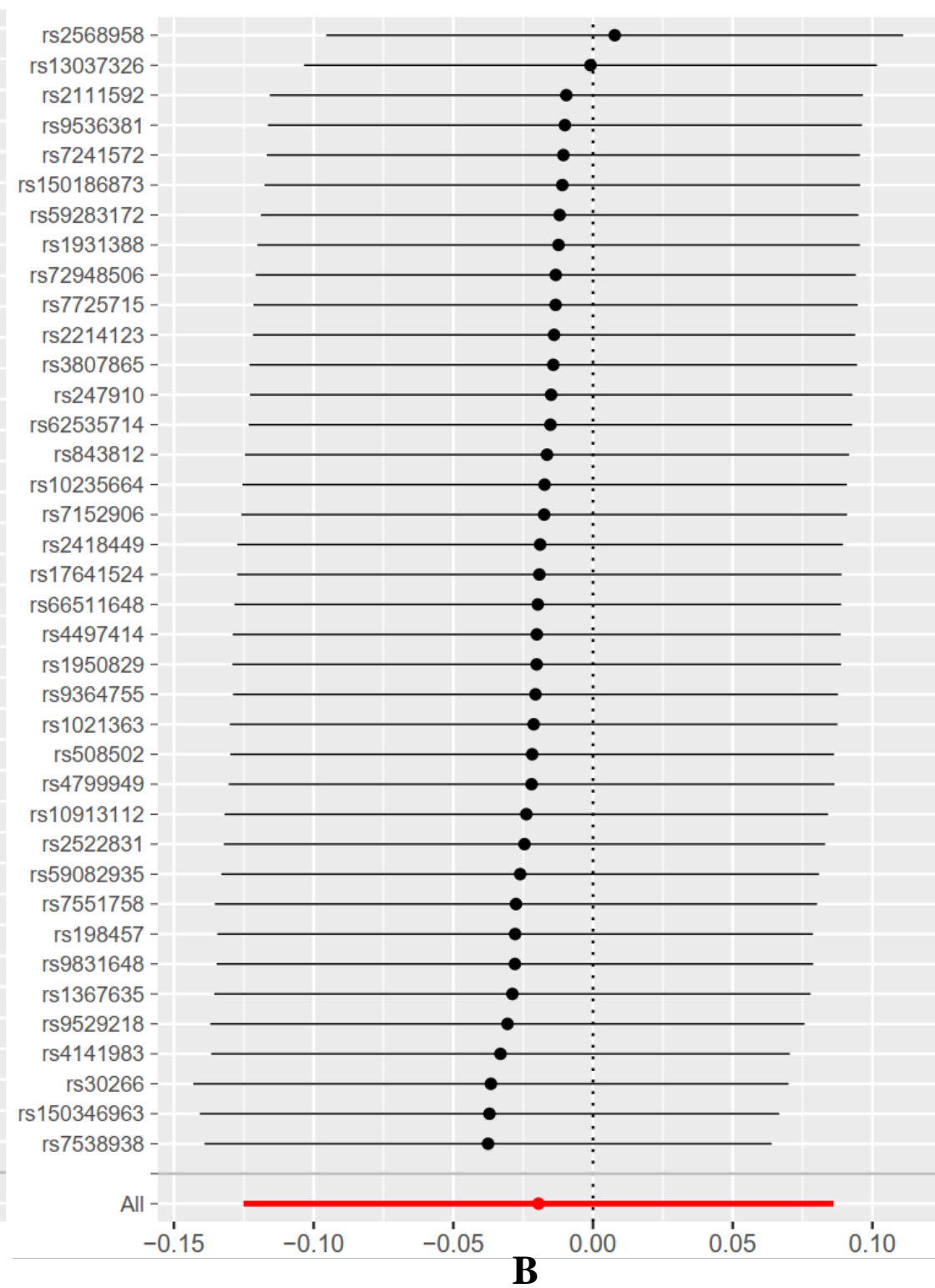

Supplement: S13 Fig — (A) All BC. (B) ER+ BC. (PDF) [file pone.0291006.s013.pdf]

## MR Test

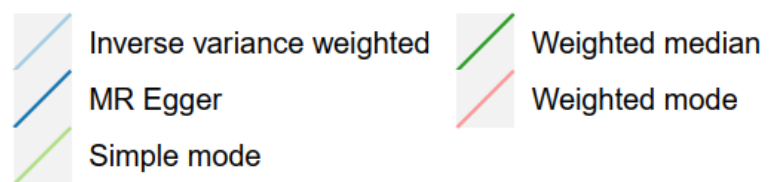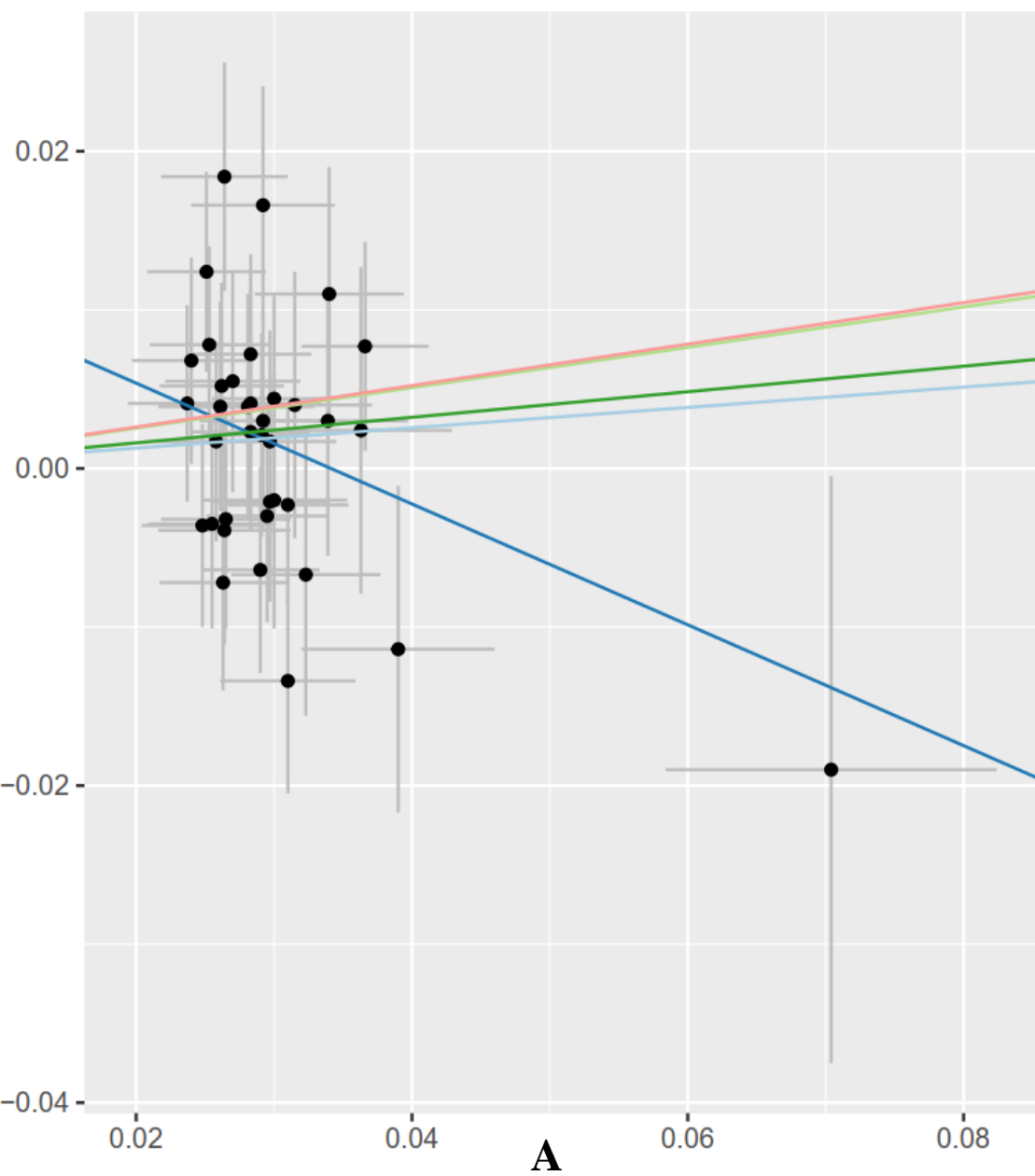

## MR Test

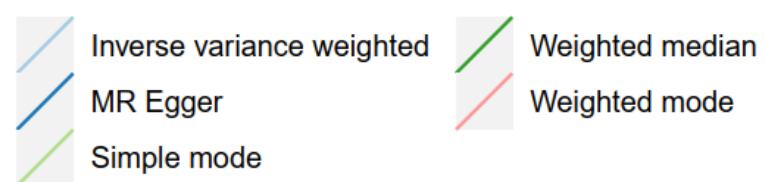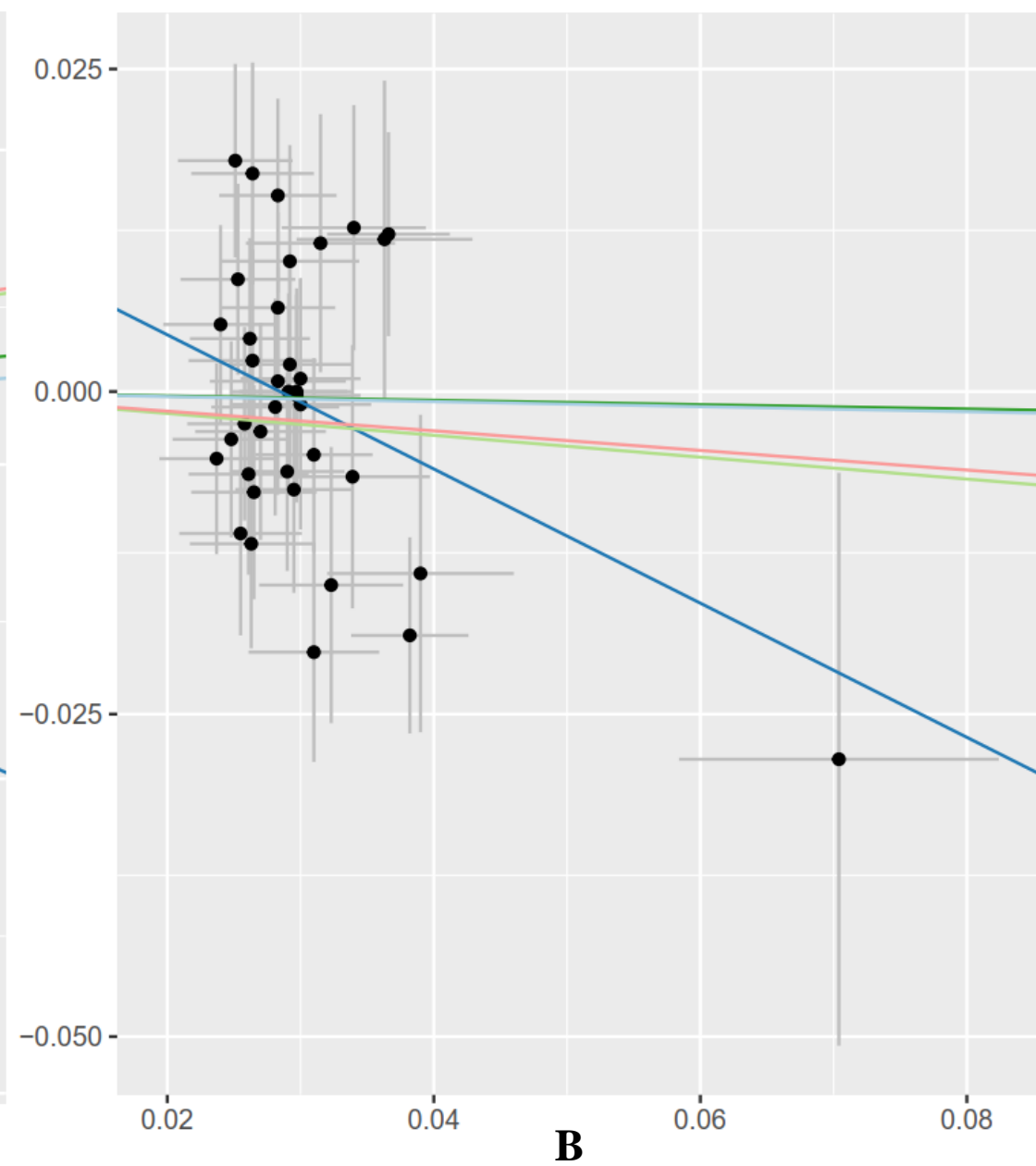

Supplement: S14 Fig — (A) All BC. (B) ER+ BC. (PDF) [file pone.0291006.s014.pdf]

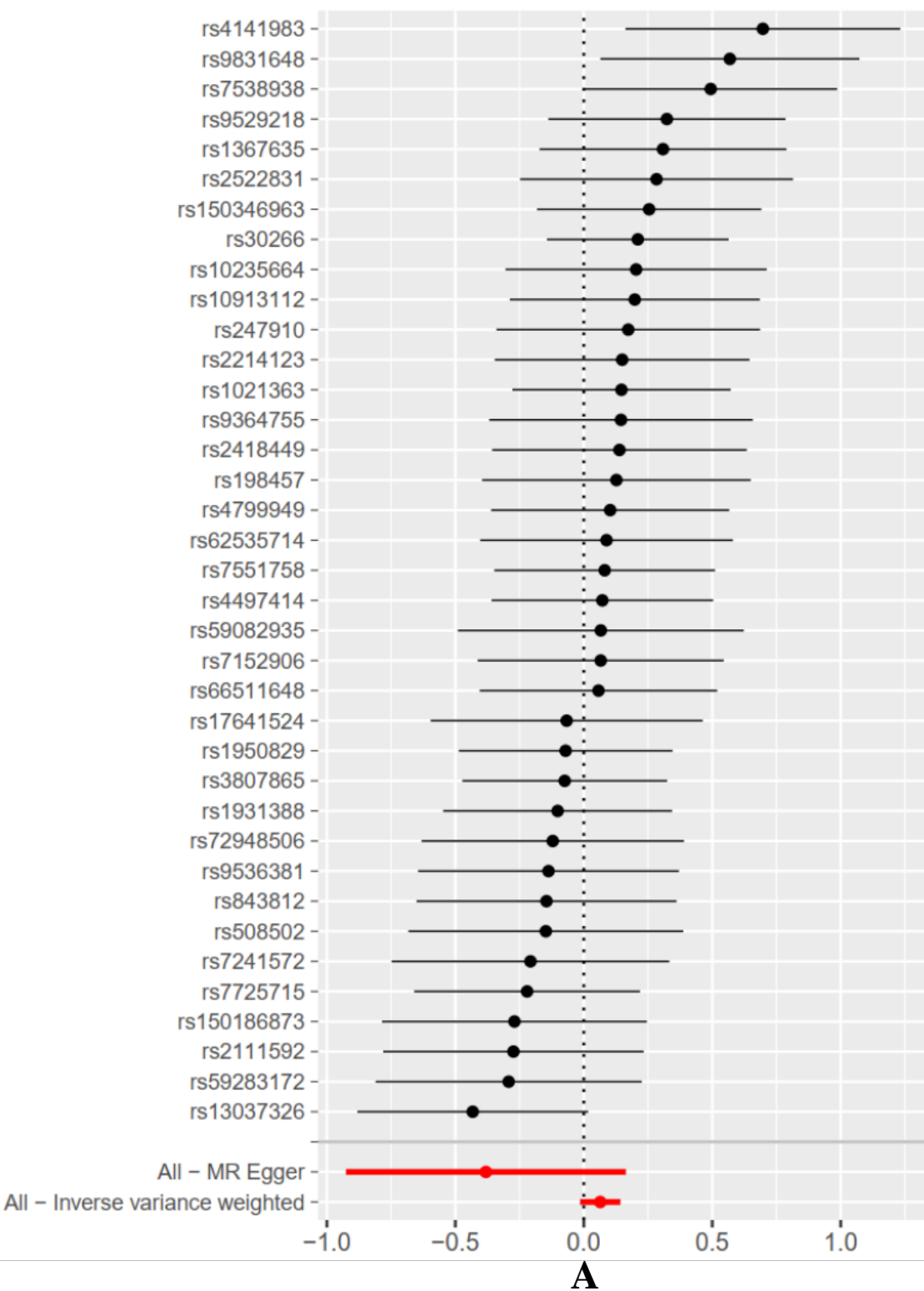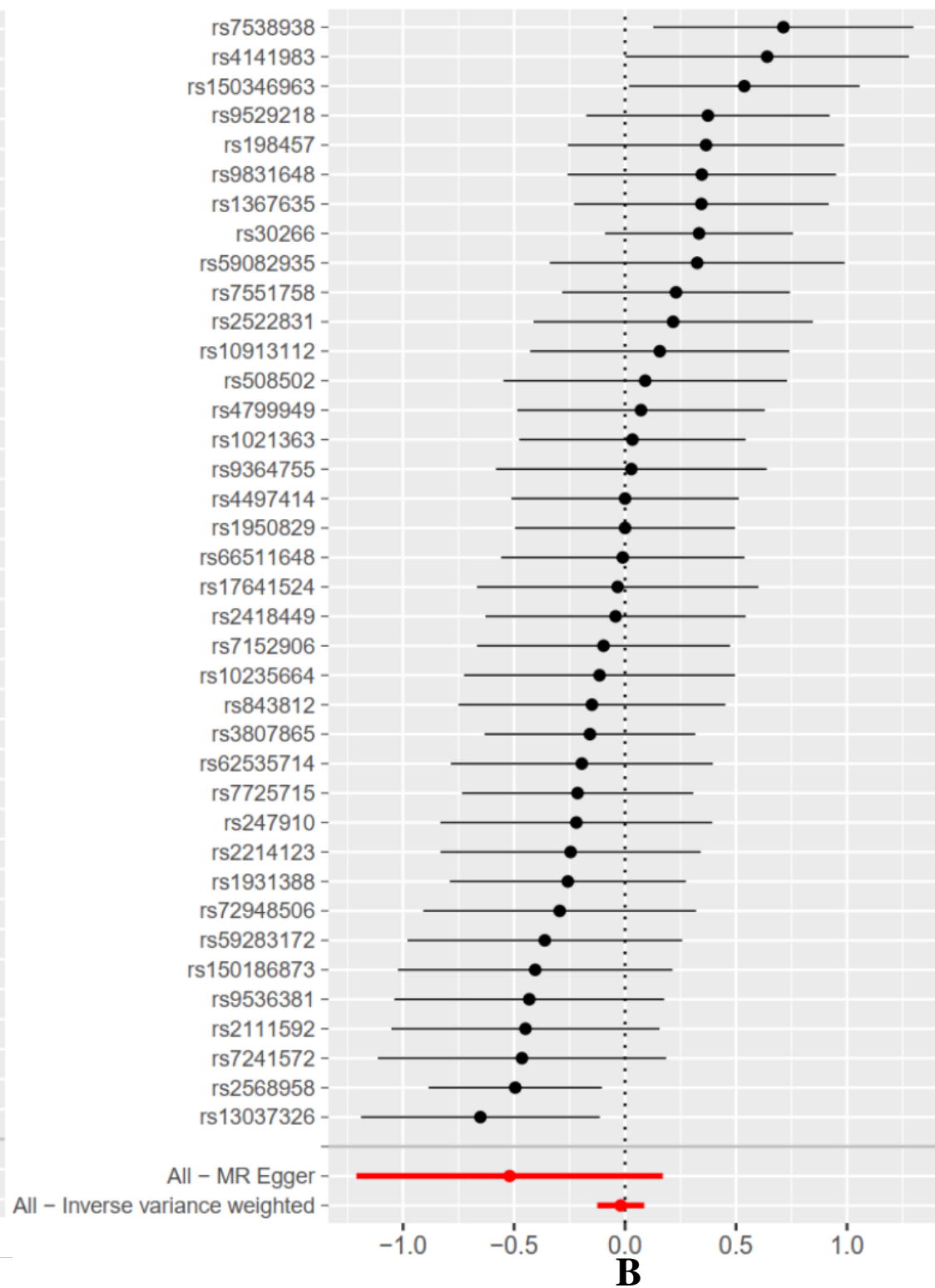

Supplement: S15 Fig — (A) All BC. (B) ER+ BC. (PDF) [file pone.0291006.s015.pdf]
