# Supplementary material for: Severe mental illness and the risk of breast cancer: A two-sample, two-step multivariable Mendelian randomization study
Source: PLoS One. 2023 Sep 1;18(9):e0291006. doi: 10.1371/journal.pone.0291006 (PMC10473543; doi:10.1371/journal.pone.0291006)
Supplement: S1 Fig — (A) All BC. (B) ER+ BC. (C) ER-BC. (PDF) [file pone.0291006.s001.pdf]

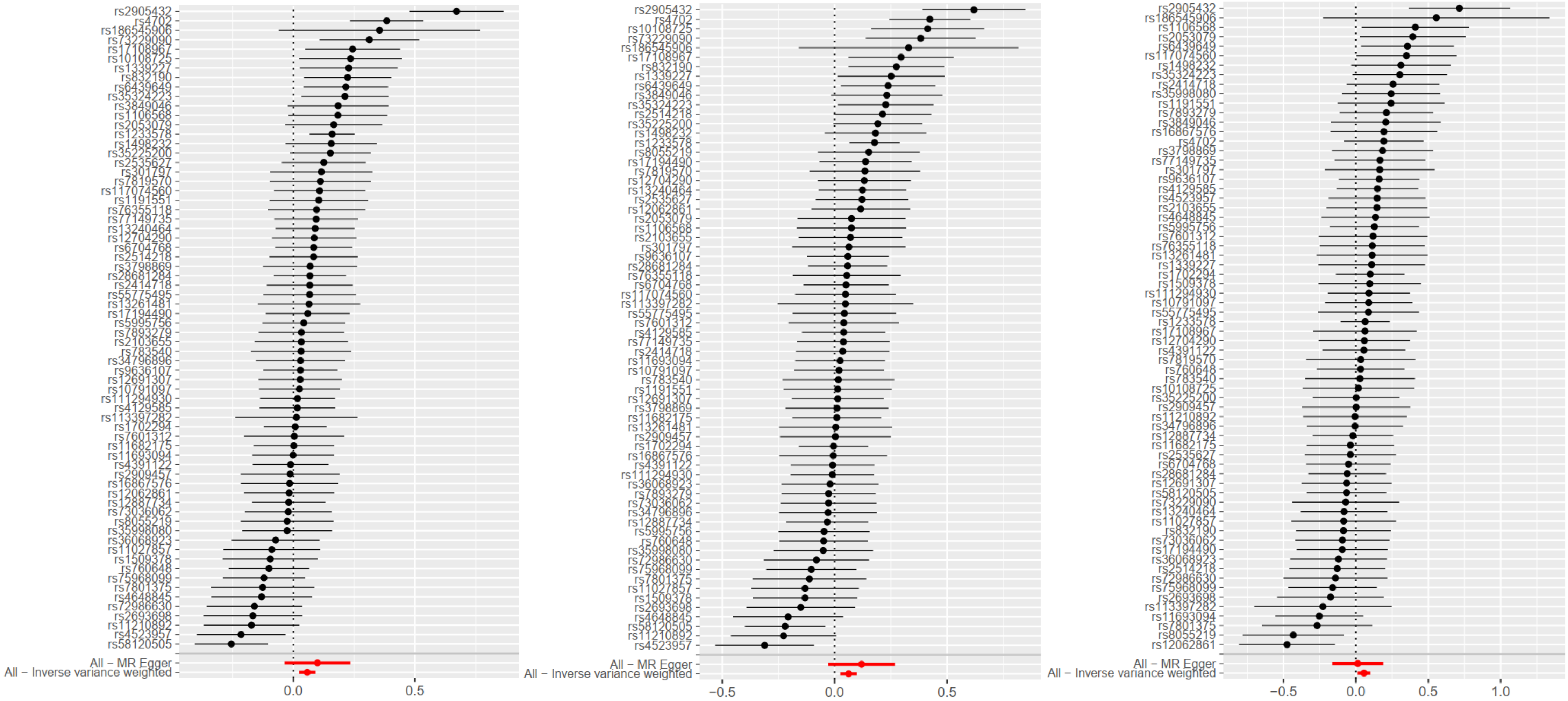
All – MR Egger
All – Inverse variance weighted
0.0
0.5
All – MR Egger
All – Inverse variance weighted
−0.5
0.0
0.5
All – MR Egger
All – Inverse variance weighted
−0.5
0.0
0.5
1.0

A

B

C
